# *BRCA1*-methylated triple negative breast cancers previously exposed to neoadjuvant chemotherapy form RAD51 foci and respond poorly to olaparib

**DOI:** 10.3389/fonc.2023.1125021

**Published:** 2023-03-17

**Authors:** Carolina Velazquez, Esin Orhan, Imene Tabet, Lise Fenou, Béatrice Orsetti, José Adélaïde, Arnaud Guille, Simon Thézénas, Evelyne Crapez, Pierre-Emmanuel Colombo, Max Chaffanet, Daniel Birnbaum, Claude Sardet, William Jacot, Charles Theillet

**Affiliations:** ^1^ Institut de Recherche en Cancérologie de Montpellier, IRCM U1194, Montpellier University, INSERM, ICM, CNRS, Montpellier, France; ^2^ Centre de Recherche en Cancérologie de Marseille, CRCM UMR1068, Aix-Marseille University, IPC, CNRS, Marseille, France; ^3^ Biometry Unit, Institut du Cancer de Montpellier, Montpellier, France; ^4^ Unité de Recherche Translationnelle, Institut du Cancer de Montpellier, Montpellier, France; ^5^ Oncological Surgery, Institut du Cancer de Montpellier, Montpellier, France; ^6^ Clinical Oncology, Institut du Cancer de Montpellier, Montpellier, France

**Keywords:** TNBC, BRCA1 methylation, RAD51, nuclear foci, HRD (homologous recombination deficiency)

## Abstract

**Background:**

About 15% of Triple-Negative-Breast-Cancer (TNBC) present silencing of the *BRCA1* promoter methylation and are assumed to be Homologous Recombination Deficient (HRD). *BRCA1*-methylated (*BRCA1*-Me) TNBC could, thus, be eligible to treatment based on PARP-inhibitors or Platinum salts. However, their actual HRD status is discussed, as these tumors are suspected to develop resistance after chemotherapy exposure.

**Methods:**

We interrogated the sensitivity to olaparib *vs*. carboplatin of 8 TNBC Patient-Derived Xenografts (PDX) models. Four PDX corresponded to *BRCA1-Me*, of which 3 were previously exposed to NeoAdjuvant-Chemotherapy (NACT). The remaining PDX models corresponded to two *BRCA1*-mutated (*BRCA1-Mut*) and two BRCA1-wild type PDX that were respectively included as positive and negative controls. The HRD status of our PDX models was assessed using both genomic signatures and the functional BRCA1 and RAD51 nuclear foci formation assay. To assess HR restoration associated with olaparib resistance, we studied pairs of *BRCA1* deficient cell lines and their resistant subclones.

**Results:**

The 3 *BRCA1*-*Me* PDX that had been exposed to NACT responded poorly to olaparib, likewise *BRCA1-WT* PDX. Contrastingly, 3 treatment-naïve BRCA1-deficient PDX (1 *BRCA1*-Me and 2 *BRCA1*-mutated) responded to olaparib. Noticeably, the three olaparib-responsive PDX scored negative for BRCA1- and RAD51-foci, whereas all non-responsive PDX models, including the 3 NACT-exposed *BRCA1-Me* PDX, scored positive for RAD51-foci. This suggested HRD in olaparib responsive PDX, while non-responsive models were HR proficient. These results were consistent with observations in cell lines showing a significant increase of RAD51-foci in olaparib-resistant subclones compared with sensitive parental cells, suggesting HR restoration in these models.

**Conclusion:**

Our results thus support the notion that the actual HRD status of *BRCA1-Me* TNBC, especially if previously exposed to chemotherapy, may be questioned and should be verified using the BRCA1- and RAD51-foci assay.

## Background

Triple-negative breast cancers (TNBCs) represent 15% of all breast cancers and its most aggressive subtype (1, 2). Despite good initial chemosensitivity, these tumors show early relapse (1, 3) and, until recently, in the absence of validated drugs only a minority of TNBC were eligible for targeted therapies, thus, stressing the need to develop novel approaches (4).

Another interesting characteristic of TNBC is that this subtype comprises the largest fraction of BRCA deficient breast tumors (5, 6). BRCA deficiency was originally shown to result from coding mutations affecting the *BRCA1* or *BRCA2* genes, which are the principal determinants of genetic predisposition to breast and ovarian cancers and play central role in Homologous Recombination (HR) Repair, also called BRCA pathway (7). HR is an essential and accurate DNA repair pathway (7, 8) and, noticeably, tumors with an HR deficiency (HRD) show elevated genetic instability (9) and accrued sensitivity to DNA cross linking agents such as platinum salts (10, 11). Accordingly, it has been proposed to include platinum in the standard of care of TNBC (12). Furthermore, over the past decade, it has been demonstrated that breast tumors with germline *BRCA1* or *BRCA2* mutations are exquisitely sensitive to PARP inhibitors, as part of a synthetic lethal interaction (13–16).

While germline *BRCA1 or BRCA2* mutations have, until recently, been the only validated indications for PARPi-based therapy in breast and ovarian cancer, it has become clear that they were not the sole causes of an HRD phenotype. Indeed, HRD has also been associated with somatic mutations and/or epigenetic silencing affecting the *BRCA1* and *BRCA2* genes, as well as other genes in the pathway, such as *PALB2*, *RAD51B*, *RAD51C* or *RAD51D* (5, 6). Because of their obvious clinical implications, the questions of the actual number of TNBC presenting HRD and the best approach to detect them have drawn increasing attention. Whole Genome Sequencing of large cohorts of breast and ovarian cancers have revealed that BRCA-deficient tumors presented specific patterns of genomic rearrangements, corresponding to scars left behind in the tumor genome by faulty repair (17). Different genomic signatures (HRDetect, HRD-score, Tandem Duplicator Phenotype, copy number signatures) were established and used to stratify TNBCs and ovarian cancers (17–20). Some of these signatures showed strong association with *BRCA1/2* mutations (germline or somatic), as well as epigenetic silencing of *BRCA1* (17–19, 21). Consequently, the fraction of TNBC with an HRD phenotype, which initially was estimated to range 2.7-17.5% (22, 23), when only germline *BRCA1/2* mutations were taken into account, raised up to 35% on the basis of genomic signatures (17–19, 21). Interestingly, tumors with an HRD genomic profiles were associated with better response to therapy and a more favorable disease outcome (24–27).

Hence, the extension of current PARPi-based therapy indications in TNBC beyond patients bearing germline *BRCA1/2* mutations has become a major question. In particular, the actual sensitivity of tumors with epigenetically silenced *BRCA1* gene due to the hypermethylation of its promoter is of particular interest, as they represent an appreciable 15 to 20% of TNBC (19, 21, 28), but has led to conflicting conclusions (27, 29–32). In particular, the actual BRCA-deficiency of post-treatment residual BRCA1-hypermethylated tumors and their subsequent recurrences has been questioned (31). This point concurs with observations made in different model systems showing that BRCA-deficient tumors rapidly acquire treatment resistance and, in most cases, resistance is due to the partial or complete restoration of HRR upon exposure to either platinum or PARPi (33, 34). Thus, the actual sensitivity of TNBC showing all signs of HRD and, particularly, those with *BRCA1* hypermethylation that have been previously exposed to chemotherapy in the course of the disease could be questioned (31, 35).

To this aim, we tested in the present study the sensitivity of 8 PDX models, comprising 7 TNBC (2 *BRCA1-WT*, 4 *BRCA1*-methylated (*BRCA1-Me*), 1 *BRCA1-Mut*) and 1 *BRCA1-Mut* High Grade Ovarian Serous Ovarian Carcinoma (HGSOC), to the PARP inhibitor olaparib and to carboplatin (CBP). Of the 8 PDX models tested, 3 showed stable disease (SD), while the 5 others progressed under olaparib. PDX models that responded to olaparib were all treatment naïve and corresponded to 2 *BRCA1*-mutated and one *BRCA1*-methylated PDX. The five non-responsive PDX comprised 2 *BRCA1* wild type and 3 *BRCA1*-methylated that, noticeably, were established from TNBC tumors that responded poorly to neo-adjuvant chemotherapy (NACT). We, thus, interrogated the HRD status of the tested PDX models and used BRCA1, RAD51, γH2AX and 53BP1 nuclear foci in olaparib treated PDX as read-outs of HR functionality and of DNA damage response. PDX models with reduced BRCA1 and RAD51 foci formation corresponded to olaparib responders, whereas non-responders were all RAD51-foci positive. Remarkably, the three *BRCA1-Me* PDX established from tumors that responded poorly to NACT were RAD51-foci-positive and progressed under olaparib. This suggested that, despite severely reduced BRCA1 protein expression, HR remained at least partially functional in these models, thus precluding their sensitivity to olaparib. We treated *BRCA1* deficient cell lines with olaparib and derived resistant variants. We observed increased BRCA1 and RAD51 foci formation in olaparib resistant variants. The data presented herein suggest that tumors with epigenetically silenced *BRCA1* that have been exposed to genotoxic treatment show poor response to olaparib and exhibit BRCA1 and RAD51 foci formation capacity compatible with HR proficiency. Our data, thus, suggest that olaparib may not be a good indication in tumors with epigenetically silenced *BRCA1* and supports the implementation of BRCA1 and RAD51 nuclear foci assay as functional read out of HR deficiency in TNBC.

## Materials and methods

### TNBC and HGSOC PDX models and in vivo treatment

TNBC and Ovarian cancer PDX models establishment was as described (36, 37). PDX models used are described in **Table 1**. The study was reviewed and approved by the ethics committees for animal experimentations of the University of Montpellier (CEEA-LR-12028). PDX models were established from fresh tumor fragments obtained from the Pathology Department at the Comprehensive Cancer Center of Montpellier (ICM) after informed consent of the patients. Establishment of PDX models was reviewed and approved by the institutional review board. Approximately 50 mm^3^ PDX fragments were grafted subcutaneously into the flank of 3-4week old Swiss-nude female mice (Charles Rivers, Saint-Germain-sur-l’Arbresle, France). The present study comprised three experimental arms; vehicle, olaparib and Carboplatin (CBP) treatment comprising 6 to 8 mice per arm. When median tumor volume reached 100-150mm^3^, mice were randomly distributed in the two arms and treatment was started. Olaparib (Lynparza, AstraZeneca) was administered orally 5 times/week for 5 weeks at 100 mg/kg. CBP (Accord Healthcare, Middlesex, UK) was administered by intra-peritoneal (IP) injection twice per week for 4 weeks at 50 mg/kg CBP. At treatment end, mice were euthanized to collect tumor samples for further biochemical (RNA and proteins) or histological analyses. Some mice were kept for tumor volume monitoring.

**Table 1:** Principal bio-clinical characteristics of the patient tumors from which the PDX models were derived.

PDX ID	BRCA1 Status	Cancer type	Grade or Stage	NACT	Type of NACT	response to NACT	RFS months	OS months
**b1995**	WT	TNBC	SBR III	No	NA	NA	>120	>120
**b3804**	WT	TNBC	SBR III	yes	Taxol+ avastin	Poor response	>120	>120
**b3977**	Me/Me	TNBC	SBR III	yes	FEC+T	refractory	5	9
**b4122**	Me/Me	TNBC	SBR III	No	NA	NA	>120	>120
**15b0018**	Me/Me	TNBC	SBR II	yes	FEC+T	Poor response	35	>60
**15b1516**	U/Me	TNBC	SBR III	yes	FEC+T	refractory	7	8
**Tm168**	Mut pS1524fs	TNBC	SBR III	No	NA	NA	3	7
**o10047**	Mut Del exon 8-13	HGSOC	Stage IV	No	NA	NA	>145	>145

WT, wild type; Me/Me both alleles methylated. U/Me hemi methylation. TNBC, triple negative breast cancer; HGSOC, High grade serous ovarian carcinoma. NC, not communicated; NA, not applicable; NACT, neoadjuvant chemotherapy; FEC, Fluorouracy. Epirubicin. Cyclophosphamide; RFS, recurrence free survival; OS, Overall Survival.

### MS-PCR

CpG methylation patterns at the *BRCA1* promoter were determined using the MS-PCR assay as previously described (28).

### Array-CGH

For each PDX sample, the genomic profile was established by using aCGH onto high-resolution 4 × 180 K CGH-microarrays (SurePrint G3-Human CGH-Microarray, Agilent Technologies). Human female DNA was used as reference (G152A, Promega, Madison, WI, USA). Both experimental and analytic methods have been previously described (38). All probes for aCGH were mapped according to the Genome Reference Consortium Human Build 37 (CGCh37/Hg19; https://www.ncbi.nlm.nih.gov/assembly/GCF_000001405.13/). We used two different threshold values (log2 ratio > 0.5 and 1.0) to distinguish low- (gain/) from high-(amplification) and (log2 ratio < -0.3 and -1) to distinguish simple loss from deletion CAN (Copy Number Alterations), respectively. Percentage of altered genome was the number of probes above the threshold divided by the total number of probes for autosomal chromosomes.

### Genetic instability and HRD scores

For each tumor, to evaluate genetic instability, we quantified the activity of the 17 copy number signatures described (20) with the R package CINSignatureQuantification.

For each tumor, a HRD score (HRDaCGH score), based on losses of heterozygosity (LOH), was calculated as previously described (39). A score ≥ 10 was considered as HRD-high. The percentage of genome altered was calculated as the sum of altered probes divided by the total number of probes after removing sexual chromosomes.

### Next generation sequencing

Mutation profiles were established by using targeted-NGS panel of 559 genes commonly mutated in breast cancer (40). The list of genes targeted is available in the Supplementary Information. The sequence data were aligned to the human reference genome (UCSC hg19) using Burrows–Wheeler Aligner (41). Tumor samples were sequenced at an average depth of 851× (range, 520 to 1055) for the targeted regions. Bam files were processed as described (40). Single nucleotide variants (SNVs) calling was performed with a consensus approach using 8 variants callers for the SNV (Freebayes) (42), HaplotypeCaller (43), LoFreq (44), Mutect2 (45), Pisces (46), Platypus (47), VarDict (48) and Varscan2 (49) and 10 variants callers for the indel (FreeBayes (42), HaplotypeCaller (43), LoFreq (44), Mutect2 (45), pindel (50), Pisces (46), Platypus (47), Scalpel (51), VarDict (48) and Varscan2 (49)). Variants called by less than 5 variants callers were filtered out. Then, the filtered variants were annotated with the Annotate Variation Software (ANNOVAR, version 2013-11-12). Known variants found in dbsnp129 and dbsnp137 with a variant allele frequency (VAF) superior to 1% (1000 G or ESP6500) were removed. Finally, low frequency SNVs and indels that were suspected to be false positives were systematically inspected with IGV version 2.3.32 (52, 53). Mutations were classified as “neutral” or “damaging” using the majority rule of predictor software (provided by dbnsfp: Sift, Polyphen2, LRT, MutationTaster, MutationAssesor, FATHMM, RadialSVM, LR) as previously described (54). A “recurrent” mutation, also called “hot spot”, was defined as being found more than 10 times in the Catalogue of Somatic Mutations in Cancer (COSMIC V68) database (http://cancer.sanger.ac.uk/cosmic).

### RT qPCR

Total RNA was isolated from cell lines lysed in TRIzol (Invitrogen, Fisher Scientific, Illkirch-Graffenstaden, France), while PDX tumors were lysed using Lysing Matrix D (MP Biomedicals™, Doornveld, France). Subsequently, the RNA was extracted using the RNeasy Kit (Qiagen, Les Ulis, France) following manufacturer instructions. cDNAs were synthesized from 1μg of total RNAs using random hexamers and SuperScript III Reverse transcription (Invitrogen, Fisher Scientific, Illkirch-Graffenstaden, France). Real-time qPCR was performed on a LightCycler 480 SW 1.5 apparatus (Roche, Meylan, France) with ONEGreen^®^ FAST QPCR PREMIX (Ozyme, Saint Cyr l’Ecole, France) and designed human specific primers (**Supplementary Table 1**). Results were quantified with a standard curve generated by serial dilutions of a reference cDNA preparation. GAPDH transcripts were used for normalization. The fold change in gene expression was calculated as: Fold change = 2^-ΔΔCT^.

### Cell lines and CRISPR-Cas9 engineered mutants

SUM159 and SUM149 TNBC cell lines a generous gift from Dr S Ethier (MUSC, Charleston, SC), were maintained in Ham’s F-12 medium (Gibco™, Fisher Scientific, Illkirch-Graffenstaden, France) supplemented with 5% FBS, 10 µg/ml insulin, 1µg/ml hydrocortisone and 1% Antibiotic-antimycotic (100X) (Gibco™, Fisher Scientific, Illkirch-Graffenstaden, France). UWB1.289PT cell line was obtained from the American Type Culture Collection (ATCC) and maintained in the 50% RPMI-1640 (Gibco™), 50% MEGM (MEGM Bullet Kit; CC-3150, Lonza, Basel, Switzerland) and supplemented with 3% FBS, 1% Antibiotic-antimycotic (100X) (Gibco™, Fisher Scientific, Illkirch-Graffenstaden, France). HCC38 cell line was obtained from ATCC and maintained in RPMI-1640 supplemented with 10% FBS and 1% Antibiotic-antimycotic.

For CRISPR-Cas9 generation of KO clones, SUM159PT cells were first transduced with a plasmid vector containing doxycycline inducible lentiviral expression of SpCas9. Lentiviral transduction was performed on 70% confluent cell cultures. Viral particles were added in the fresh medium containing 8µg/ml polybrene. After 16h the medium was changed and 2µg/ml puromycin added for cell selection for at least 5 days. Next, the cells were transduced as described above with two lentiviral plasmid vectors for the expression of sgBRCA1 (kind gift from Yea-Lih Lin, IGH, Montpellier). After lentiviral transduction, cells were selected with 400µg/ml G418 for 10 days and Cas9 expression was induced by treating a population of cells with 1µg/ml of doxycycline for 6 days. The cells were then cloned and clones verified for the KO by Sanger sequencing and western blot. All cell lines and selected clones were genetically typed by Eurofins Genomics cell line authentification (Eurofins Genomics, Les Ulis).

### Protein extraction and Western blotting

Protein extracts were prepared by lysing either tumor tissue or cell line pellets on ice for 30 min in Tris-HCl pH7.4 50mM, NaCl 100mM, NaF 50mM, β-glycerophosphate 40mM, EDTA 5mM, Triton X100 1%, Aprotinin 10mg/ml, PMSF 100mM, Leupeptin 1mM, Pepstatin 1mM, followed by a short centrifugation to pellet debris. Protein concentrations were measured using the BCA kit (Fisher Scientific, Illkirch-Graffenstaden, France) SDS-PAGE gel electrophoresis was done on 30µg protein samples subsequently transferred onto nitrocellulose membranes (Amersham, Velizy-Villacoublay, France) and incubated overnight at 4°C with the primary antibody. Antibodies used are listed in a separate section. Membranes were then washed and incubated with the appropriate secondary antibody in 5% non-fat dry milk in PBST for 2h at room temperature and revealed by incubation with Chemiluminescent HRP Substrate (Sigma Aldrich, Saint Quentin Fallavier, France).

### Immunofluorescence

For cell lines, cells were grown on 12mm diameter slides cover slips in 24 well-plate for 24h, then drugs were added at the predetermined IC50 concentration. After 24h drugs were washed off and cells prepared as described below. For tumor tissues, 6μm cryosections were prepared from OCT embedded deep frozen tissue and mounted on Fisherbrand™ Superfrost™ Plus Microscope Slides (Fisher Scientific, Illkirch-Graffenstaden, France) and stored at -80°C until used. Cells and tumor sections were sequentially subjected to mild extraction (0.4% Triton in PBS, 5min in cold), fixation (4% PFA diluted in PBS) and blockage/permeabilization (3% BSA + 0.2% Triton in PBS, 1 hour at room temperature), incubated overnight at 4°C with the primary antibody (diluted in 3% BSA + 0.2% Triton in PBS), then with the secondary antibody (diluted in 3% BSA + 0.2% Triton in PBS, 1h at room temperature). Between each step, slides were washed 3 times with PBS. Tumor cryosections were immersed 0.1% SBB (Sigma Aldrich, Saint Quentin Fallavier, France) and 70% ethanol for 20 minutes at room temperature to reduce tissue autofluorescence and subsequently washed three times for 5 minutes PBS with 0.02% Tween 20. Stained sections or cell were counterstained with DAPI (Fisher Scientific, Illkirch-Graffenstaden, France) to stain the nuclei and cover slips were mounted with MWL4-88 (Citifluor, CliniSciences, Nanterre, France) and stored at 4°C. Antibodies used are described in the Antibody section.

Immunofluorescence images were acquired using Zeiss microscope with a 63X-immersion oil lens and generated using Zeiss Blue software. RAD51, BRCA1, 53BP1 and γH2AX nuclear foci were scored using the CellProfiler image analysis software (version 2.2.0, Broad Institute). At least three biological replicates of each model (both vehicle- and olaparib-treated) were analyzed. Cells presenting >5 foci/nucleus for RAD51, BRCA1, 53BP1 or γH2AX were considered positive and tumors presenting >10% of positive cells were scored positive for the given marker.

### Antibodies

Immunofluorescence; rabbit anti-RAD51 PC130 1:300 (Merck Millipore Sigma Aldrich, Saint Quentin Fallavier, France), rabbit anti-geminin 52508 1:200 (CST OZYME, Saint Cyr l’Ecole, France), mouse anti-BRCA1 sc-6954 1:100 (SCBT, Heidelberg, Germany), mouse anti-γ-H2AX (H2-3F4, kind gift from Dr. Mustapha Oulad-Abdelghani, MAB-IGBMC Illkirch-Graffenstaden, 1:4000), rabbit anti-53BP1 NB100-304 1:500 (Bio-techne LTD, Abington, UK). Secondary antibodies; goat anti-mouse Alexa Fluor 488 (Abcam ab150113, 1:1000), goat anti-rabbit Alexa Fluor 555 ab150078 1:1000 (Abcam, Cambridge, UK).

Western blotting; BRCA1 9010 1:500 (CST OZYME, Saint Cyr l’Ecole, France), BRCA2 A303-434A 1:1000 (Bethyl OZYME, Saint Cyr l’Ecole, France), PARP1 WH0000142M1 1:1000 (Sigma Aldrich, Saint Quentin Fallavier, France), RAD51 8875, 1:1000 (CST OZYME, Saint Cyr l’Ecole, France) and alfa tubulin T9026 1:20000 (Sigma Aldrich, Saint Quentin Fallavier, France); secondary antibodies goat anti-mouse-HRP 70745 1:10000 (CST OZYME, Saint Cyr l’Ecole, France) and goat anti-rabbit-HRP 7076 1:10000 (CST OZYME, Saint Cyr l’Ecole, France).

## Results

### PDX models show variable response to olaparib

To test the relative sensitivity of TNBC with silenced *BRCA1* due to promoter hypermethylation (designated hereafter *BRCA1*-*Me*) to the PARP inhibitor olaparib and assess the impact of neoadjuvant treatment (NACT) on olaparib sensitivity, we selected 8 PDX models (7 TNBC and 1 High Grade Serous Ovarian Carcinoma) showing different *BRCA1* profiles. Our experimental PDX set comprised 2 *BRCA1* wild type (*BRCA1-*WT) TNBC (b1995, b3804), 4 *BRCA1-Me* TNBC (b3977, b4122, 15b1516, 15b0018) and 2 (1 TNBC and 1 HGSOC) *BRCA1* mutated (*BRCA1*-Mut) models (tm168, o10047) (**Table 1**; **Supplementary Figure 1**). *BRCA1*-Me PDX models 15b1516, 15b0018 and b3977 were established from post-NACT residual tumors that had shown poor response to neoadjuvant treatment (**Table 1**; **Supplementary Figure 1**). The two *BRCA1*-*Mut* models (tm168, o10047) and the remaining BRCA1-Me model (b4122) were established from treatment naïve tumors. Hypermethylation of the *BRCA1* promoter region was determined in both the PDX models and the patient tumors using methylation specific PCR (MS-PCR). It is of note that no pre-treatment biopsy was available for the tumors that had undergone NACT, hence, *BRCA1* promoter methylation was determined after NACT on these tumor samples. Of the four *BRCA1*-*Me* PDX, 3 presented homozygous (Me/Me) methylation (15b0018, b3977, b4122) and 1 hemizygous (Me/U) methylation (15b1516) (**Supplementary Figure 2).**

**Figure 1:**
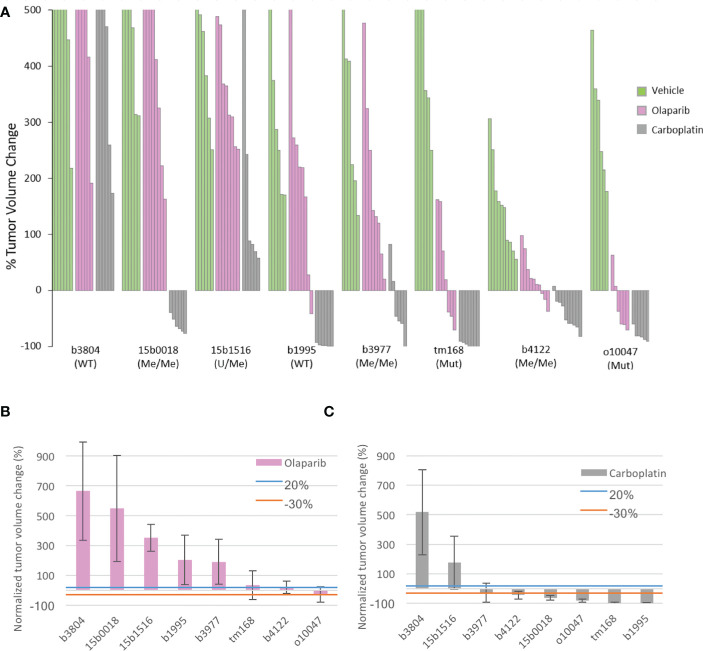
Tumor volume change of the 8 PDX models under olaparib and Carboplatin treatment and principal characteristics related to HR of the PDX models **(A)** Waterfall plot of tumor volume changes in the 3 experimental arms were computed at the end of treatment for individual tumors as the percentage of the starting volume (green vehicle; pink olaparib, grey Carboplatin) **(B)** mean tumor volume change in each PDX model treated with olaparib. **(C)** mean tumor volume change in each PDX model treated with Carboplatin. Blue an orange lines indicate the +20% and −30% thresholds for tumor response defined by the RECIST criteria; > 20% progressive (PD). < 20%- > -30% stable disease (SD). < 30% responsive (R).

PDX were grafted subcutaneously on Swiss-nude mice and olaparib was administered orally at 100 mg/kg, 5days per week for 5 weeks. In a parallel treatment group, mice received 50mg/kg Carboplatin (CBP) by intraperitoneal injection twice a week for 4 weeks, while the control group received daily the olaparib vehicle administered orally. Tumor volumes were measured twice a week. Three (tm168, b4122, o10047) of the 8 PDX showed disease stabilization or limited tumor size reduction under olaparib treatment, while the 5 other models (b3804, 15b0018, 15b1516, b1995, b3977) progressed (**Figures 1A, B**; **Supplementary Figure 3A**). Of the 3 olaparib responders, PDX tm168 and o10047 were *BRCA1*-*Mut* and b4122 was *BRCA1*-*Me*, but established from a treatment naïve tumor. The 3 other *BRCA1*-*Me* models (15b0018, 15b1516 and b3977, which progressed under olaparib, were established from tumors that had received NACT. In the CBP arms we globally noted more favorable response patterns, with 6 of 8 PDX models showing partial to complete response and 2 PDX (b3804, 15b1516) progressing under treatment (**Figures 1A, C**; **Supplementary Figure 3A**). Tumor growth was monitored after treatment end in 3 PDX, 2 *BRCA1*-*Me* TNBC (b3977, b4122) and 1 *BRCA1*-*Mut* HGSOC (o10047) (**Supplementary Figure 3B**) Both *BRCA1*-*Me* TNBC resumed growth shortly after end of olaparib administration. By contrast, the *BRCA1*-*Mut* o10047 exhibited complete regression 25 days after treatment ended and recurred after a lull of 3 weeks. In the CBP arm, progression after treatment end was observed in 1 of 3 PDX (b4122), while the two other models did not recur during the follow up period (**Supplementary Figure 3B**).

### Genomic profiles and genetic instability scores of the tested TNBC models

**Figure 2:**
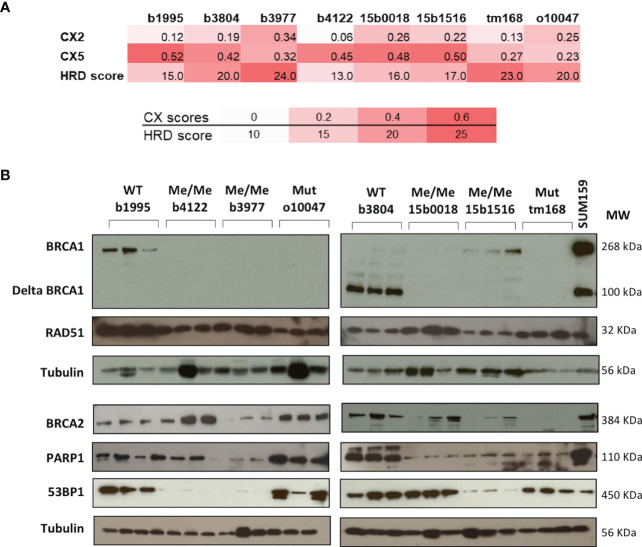
Principal characteristics related to HR of the PDX models. **(A)** CX Chromosomal instability and HRD scores of each PDX model. Color shades indicate from white to red the level of instability. **(B)** Protein expression patterns were assessed by Western blotting. The *BRCA1* status of each model is indicated on top of the autoradiograms. WT wild type. Me/Me homozygous methylation. U/Me hemizygous methylation. Mut coding mutation. For each PDX model three independent extracts from individual PDX tumors were loaded and analyzed. Proteins analyzed are indicated on the left and Molecular Weights in KDalton on the right. The protein extract of SUM159 cell line extract was added to the BRCA1 panel to illustrate the full length and the Δ11 BRCA1 protein variants.

Targeted sequencing was performed on the 7 TNBC PDX to search for mutations affecting principal DNA damage response genes and targetable cancer genes. The HGSOC PDX was analyzed by exome sequencing as part of a previous study (55). Mutations were found in the *TP53*, *PTEN*, *KRAS*, *RIF1* and *STK11* genes (7, 2, 1, 1 and 1 PDX respectively) (**Supplementary Table 2**). No mutations were detected in further DNA repair genes such as *BRCA2*, *PALB2* or the *RAD51* orthologs *RAD51B*, *C* or *D*. The 8 PDX models were also analyzed for copy number changes by array-CGH. Genetic instability and HRD scores were determined (**Figure 2A**). All TNBC models, irrespective of *BRCA1* mutation or epigenetic silencing, presented elevated genetic instability scores (CX2, CX5) shown to be linked with BRCA-deficiency (20). We also noted that all tested TNBC models presented elevated HRD scores as defined by Abkevich and coworkers (39). Thus, elevated genetic instability and HRD scores suggested preexisting HR deficiency in the PDX models used in this study, including the two *BRCA1*-*WT* models.

We took advantage of the CGH analysis to determine copy number changes affecting key repair genes in the TNBC models. Except b3804, most PDX presented hemizygous losses affecting 3 to 4 HR genes of a list including *BRCA1*, *BRCA2, PALB2, RAD51, RAD51B* and *RAD51C* suggesting that the combined copy number reductions on these key HR genes could have contributed to a global HR attenuation and elevated genetic instability in these tumors (**Supplementary Figure 4**). But we cannot exclude the existence of undetected genetic or epigenetic anomalies affecting genes involved in HR maintenance.

### Principal genes of the BRCA pathway showed protein expression profiles concordant with olaparib response

To determine the impact of promoter hypermethylation or coding mutations on BRCA1 protein expression, BRCA1 protein levels were measured by western blotting (WB) in the 8 PDX models. Noticeably, *BRCA1*-*Me* PDX b4122, b3977, 15b0018 and *BRCA1*-*Mut* o10047, tm168 showed no detectable BRCA1 band, suggesting a loss of BRCA1 functionality in these tumors. By contrast, the *BRCA1*-*WT* b1995, b3804 and the *BRCA1*-hemimethylated 15b1516 PDX presented detectable BRCA1 bands (**Figure 2B**). We noted that PDX b3804 expressed high levels of a short 100 kD BRCA1 protein isoform and low levels of the full length (268 kD) protein (**Figure 2B**). The 100 kD band is compatible with the size of the hypomorphic Δ11-BRCA1 isoform, which corresponds to a *BRCA1* splice-variant where exon 11 is excluded and that is frequently expressed in tumors bearing mutations in exon 11 (56). However, no *BRCA1* coding mutation, that could have explained the expression of the Δ11-BRCA1 isoform, was detected in PDX b3804 (**Supplementary Table 1**). We also analyzed expression of the BRCA2, RAD51, PARP1 and 53BP1 proteins, which are important actors of HR. Whereas RAD51 expression appeared elevated and relatively constant in the different PDX models, that of BRCA2 was more variable, with no obvious link with BRCA1 status or olaparib response, except possibly the *BRCA1*-*Mut* PDX tm168, which showed very low BRCA2 protein levels (**Figure 2B**). PARP1 protein expression was detected in all PDX models, but, interestingly, the lowest levels were detected in the 3 *BRCA1*-*Me* (b3977, 150018 and 15b1516) that had been exposed to NACT and responded poorly to olaparib (**Figure 1A**). Finally, 53BP1 protein was expressed at high levels in 5 models and at low levels in 3 other PDX (**Figure 2B**).

### BRCA1, RAD51 and 53BP1 nuclear foci formation in treated tumors correlate with olaparib response

**Figure 3:**
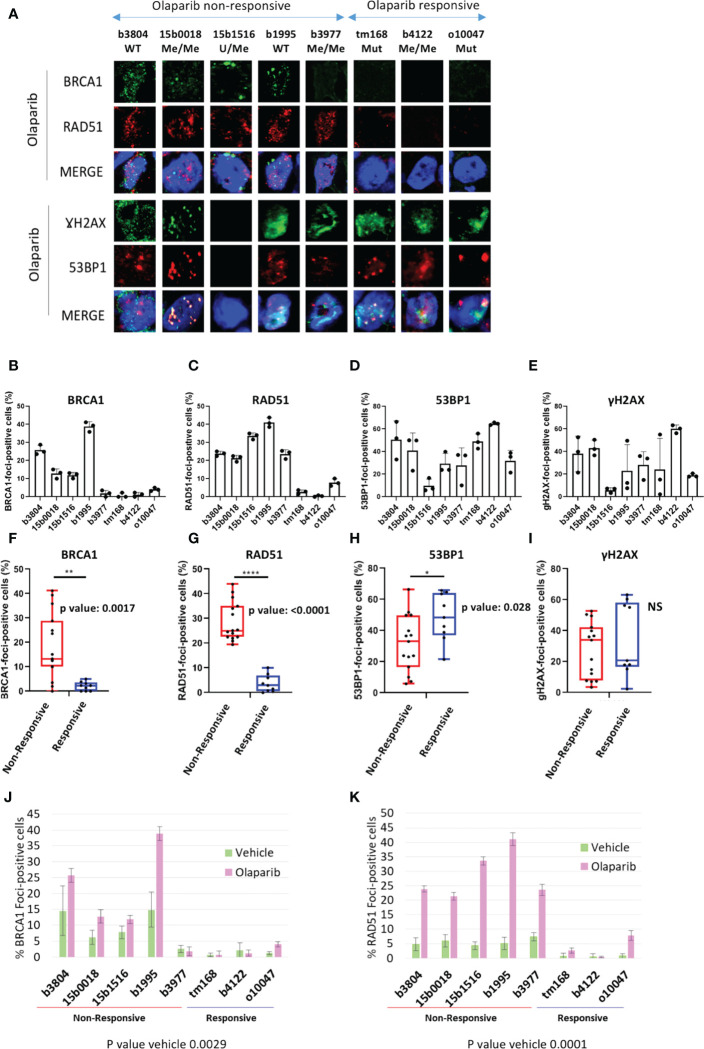
BRCA1, RAD51, γH2AX and 53BP1 nuclear foci formation in olaparib treated PDX. **(A)** Representative immunofluorescence images of frozen PDX tissue section harvested from mice sacrificed after the last administration of olaparib or before the tumor reached ethical size for vehicle treated models. PDX tumor sections were ordered from worst to best olaparib response. A complete version including vehicle treated tumors is visible in **Supplementary Figure 5**. **(B)** Quantification of BRCA1, **(C)** RAD51, **(D)** γH2AX, **(E)**: 53BP1 foci formation in olaparib treated PDX. Results are represented as % of foci positive cells in the analyzed tumor sections in each PDX. Cells presenting at least 5 foci/nucleus were considered positive. At least 100 cells were quantified in each tissue section. **(F–I)**: by two-tailed unpaired t-test analyses of correlation between foci numbers and olaparib response. Nuclear foci analyzed are indicated on top of each graph, as well as p-values. *, **,**** on top of the graph indicate the level of significance of the t-test **(J, K)** Histograms showing the percentage of BRCA1- **(J)** and RAD51-**(K)** foci positive cells of BRCA1 and RAD51 nuclear foci quantification in vehicle and olaparib treated PDX.

In HR-proficient cells, BRCA1 and RAD51 proteins cluster at DNA damage sites forming nuclear foci that can be detected by immunofluorescence labeling. Absence of BRCA1 and/or RAD51 foci is considered as a sign of HR-deficiency. We scored the fraction of cells positive for BRCA1 and RAD51 foci in cryosections of PDX models sampled at the end of olaparib and vehicle treatment and searched for an association with olaparib response. Tumors presenting >10% cells showing >5 BRCA1 or RAD51 per nucleus were scored positive. PDX sections were immunolabeled with commercial antibodies directed against BRCA1, RAD51, and the S phase marker Geminin. Because of secondary antibodies species compatibility, BRCA1 and RAD51 were co-immunolabeled (**Figure 3A**; **Supplementary Figure 5**), whereas Geminin staining was performed separately to ascertain the presence of S phase cells in each sample. Immunolabeling scoring was performed in 3 independent PDX tumors per model on at least 100 cells per section. Geminin staining showed 10 to 30% of Geminin-positive cells, confirming that tissue section actually comprised S phase tumor cells (**Supplementary Figure 6**). Four (4) of the 8 olaparib treated PDX, corresponding to 2 *BRCA1*-*Mut* (tm168, o10047) and 2 *BRCA1*-*Me* (b3977, b4122), scored negative for BRCA1-foci (**Figures 3B, J**), in agreement with the absence of BRCA1 band in the WB analysis (**Figure 2B**). BRCA1-foci positive models comprised two *BRCA1*-*WT* (b3804, b1995) and two *BRCA1*-*Me* PDX (b15b1516, 15b0018). Three of the four BRCA1-foci negative PDX (tm168, o10047, b4122) scored negative for RAD51-foci (**Figures 3A, C, J, K**) and corresponded to the 3 models that responded to olaparib (**Figures 1A, B**). Noticeably, PDX b3977, which progressed under olaparib (**Figures 1A, B**), scored positive for RAD51-foci, despite the fact it did not show BRCA1-foci (**Figures 3A, C, J, K**). All other RAD51-foci-positive PDX scored also positive for BRCA1 foci and showed mediocre response to olaparib (**Figures 1A, B**; **3A–C, J, K**). Noticeably, the 3 *BRCA1*-*Me* models established from NACT treated TNBC (15b0018, 15b1516, b3977) were RAD51-foci positive and showed scores similar to those observed in the 2 *BRCA1*-*WT* models (b1995, b3804) (**Figures 3B, J**). By contrast, the *BRCA1*-*Me* PDX b4122, which was not exposed to NACT prior PDX establishment, was both BRCA1 and RAD51-foci-negative and did not progress under olaparib. These data suggested that while b4122 was indeed HR deficient, the 3 NACT treated *BRCA1*-*Me* models were, at least partially, HR proficient.

We verified whether BRCA1 or RAD51 nuclear foci formation was associated with olaparib response (**Figures 3F-I**). We noted a significantly association of BRCA1 and RAD51 foci absence (or low levels) with olaparib response (t-test p=0.0017 and p<0.0001 respectively) (**Figures 3F, G**). Interestingly, the differences in foci positive cells between responsive and non-responsive cells were discernible in both tumor sections from olaparib treated (**Figures 3F, G**) and sections from vehicle treated tumors and showed equivalent statistical significance (**Figures 3J, K**).

We also scored γH2AX and 53BP1 foci, which are standard DNA damage markers, and observed that models presented 20 to 60% foci-positive cells for either marker, indicating severe DNA damage in olaparib treated PDX, to the exception of 15b1516 where only 5 to 10% cells scored positive (**Figures 3D, E**). Interestingly, we noted that 53BP1-foci tended to be more frequent in olaparib responsive PDX, compared with non-responsive PDX (t-test p=0.028) (**Figure 3H**). However, no difference was found with γH2AX-foci (**Figure 3I**).

### Acquired olaparib resistance in BRCA1 deficient cell lines is associated with increased BRCA1 and RAD51 foci formation and reduced levels of DNA damage

**Figure 4:**
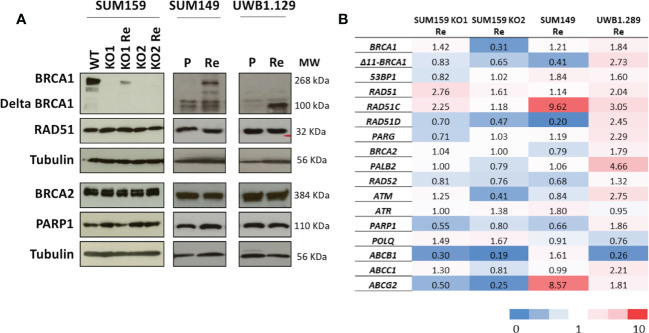
Protein and mRNA expression profiles of principal HR genes in the cell line models **(A)** Western blots showing protein expression patterns; analyzed proteins are indicated on the left and molecular weights in KDa on the right. **(B)** mRNA expression changes of genes potentially associated with olaparib resistance in cell line models selected for acquired olaparib resistance. mRNA levels are expressed as fold changes of the expression levels measured between the olaparib-resistant cell line and their parental lines used as reference (horizontal line). Reference cell lines were SUM159-KO1, SUM159-KO2 parental SUM149 and parental UWB1.289.

It is well documented that BRCA-deficient tumors or cell lines rapidly acquire resistance to treatment associated with the restoration of HRR (56). We were, thus, interested to explore the restoration of RAD51-foci formation in *BRCA1*-deficient cell lines with acquired olaparib resistance. We, thus, isolated olaparib resistant clones from three cell lines; SUM159-KO1 and SUM159-KO2, two CRISPR *BRCA1* knock out clones we engineered from the TNBC SUM159 cell line, the SUM149 TNBC cell line which bears a frameshift mutation in exon 11 (2288delT) (57) and the UWB1.289 ovarian cancer cell line also showing a frameshift mutation in exon 11 (2594delC) (58). Resistance was obtained by exposing cell cultures to incremental olaparib concentrations for at least 12 weeks. Olaparib resistant cell lines were designated with the suffix Re (SUM159-KO1-Re, SUM159-KO2-Re, SUM149-Re and UWB1.289-Re). We characterized BRCA1, BRCA2, RAD51 and PARP1 protein expression by WB, as well as RNA expression changes associated with the acquisition of olaparib resistance (**Figures 4A, B**). In SUM159-KO1 and SUM159-KO2 no BRCA1 protein was detected. SUM159-KO1-Re reexpressed the full length BRCA1 protein and SUM159-KO2-Re showed no difference in BRCA1 protein expression compared with it clone of origin. SUM149 and UWB1.289 expressed no full length BRCA1 and variable levels of the Δ11-BRCA1 100 kD band. Interestingly, SUM149-Re expressed the BRCA1 full length and increased levels of the Δ11-BRCA1 variant, whereas UWB1.289-Re showed strongly increased Δ11-BRCA1 levels (**Figure 4A**). No significant difference was found for BRCA2, RAD51 and PARP1 protein expression in the different cell line models. At the RNA expression level, we noted increased gene expression of *RAD51* and *RAD51C* in SUM159-KO1, of *RAD51C* and *ABCG2* in SUM149-Re, as well as of Δ11*-BRCA1, RAD51C, RAD51D, PALB2* and *ATM* in UWB1.289-Re which could be related with their acquisition of olaparib resistance.

**Figure 5:**
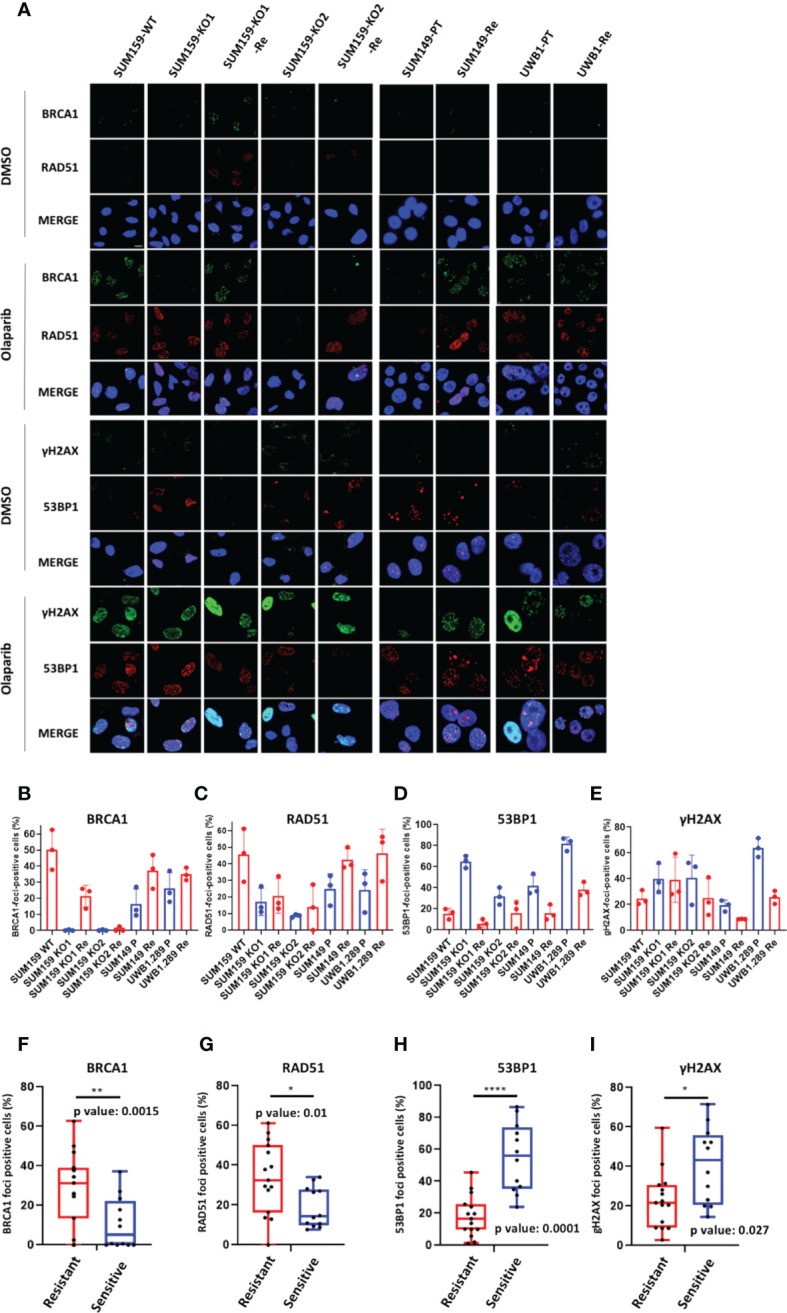
BRCA1, RAD51, γH2AX and 53BP1 nuclear foci in BRCA1 deficient cell line models **(A)** Representative immunofluorescence images of cell line models treated or not (control) with Olaparib for 24h. Cell line names are indicated on top *BRCA1*-WT (WT), BRCA1-KO (KO), BRCA1-KO-Ola-Resistant (Re), SUM149; parental (PT), Ola-Resistant (Re), UWB1.289; parental (PT), Ola-Resistant (Re) **(B)** BRCA1, **(C)** RAD51, **(D)** 53BP1, **(E)** γH2AX foci formation in the respective olaparib treated cell lines. Results are represented as % of foci positive cells in the analyzed tumor sections in each PDX. Cells presenting at least 5 foci/nucleus were considered positive. At least 150 cells were quantified in each section. Representative IF images are shown in the **Supplementary Figures**. **(F–I)**: Box plot presenting the difference in the number of BRCA1- **(F)**, RAD51- **(G)**, 53BP1-**(H)** and gH2AX- **(I)** foci positive cells between olaparib resistant and sensitive cell lines. p-values were calculated with two-tailed unpaired t-test. *, **,**** on top of the graph indicate the level of significance of the t-test.

Next, we scored BRCA1, RAD51, 53BP1 and γH2AX foci formation in the olaparib treated cell lines (**Figure 5A**). Except in SUM159-KO2-Re which does not express BRCA1, nor ∆11-BRCA1, and scored BRCA1-foci-negative, BRCA1-foci scores tended to increase in olaparib resistant clones compared with cells or origin (**Figure 5B**). A similar trend was noticeable for RAD51-foci, whose numbers nearly doubled in olaparib resistant cells relative to cell lines of origin (**Figure 5C**). By contrast, 53BP1 and γH2AX foci numbers clearly decreased in olaparib resistant variants (**Figures 5D, E**). These differences in foci numbers between olaparib resistant cells and their original counterparts, increase for BRCA1 and RAD51, decrease for 53BP1 and γH2AX, were all statistically significant and in coherence with findings we made on PDX (**Figures 5F-I**). The reduction of γH2AX-foci associated with the increase of BRCA1 and RAD51-foci observed in resistant cells suggest a global decrease of DNA damage in these cells upon olaparib treatment due to restored HR capacity. We also derived olaparib resistant cells from the hemimethylated *BRCA1-Me/UM* HCC38 TNBC cell line, whose methylation status we confirmed. While BRCA1-foci scores only modestly increased in olaparib treated cells, the fraction of RAD51-foci positive cells doubled in HCC38-Re variant cells (**Supplementary Figure 7**). Overall, our cell line data strongly support the notion that resistance to treatment and particularly to olaparib in BRCA1-deficient cancer cells is frequently associated with restoration of RAD51 foci formation, thus, signing for restored HR capacity.

### BRCA1, RAD51, γH2AX and 53BP1 nuclear-foci as predictors of the response to olaparib or carboplatin

**Figure 6:**
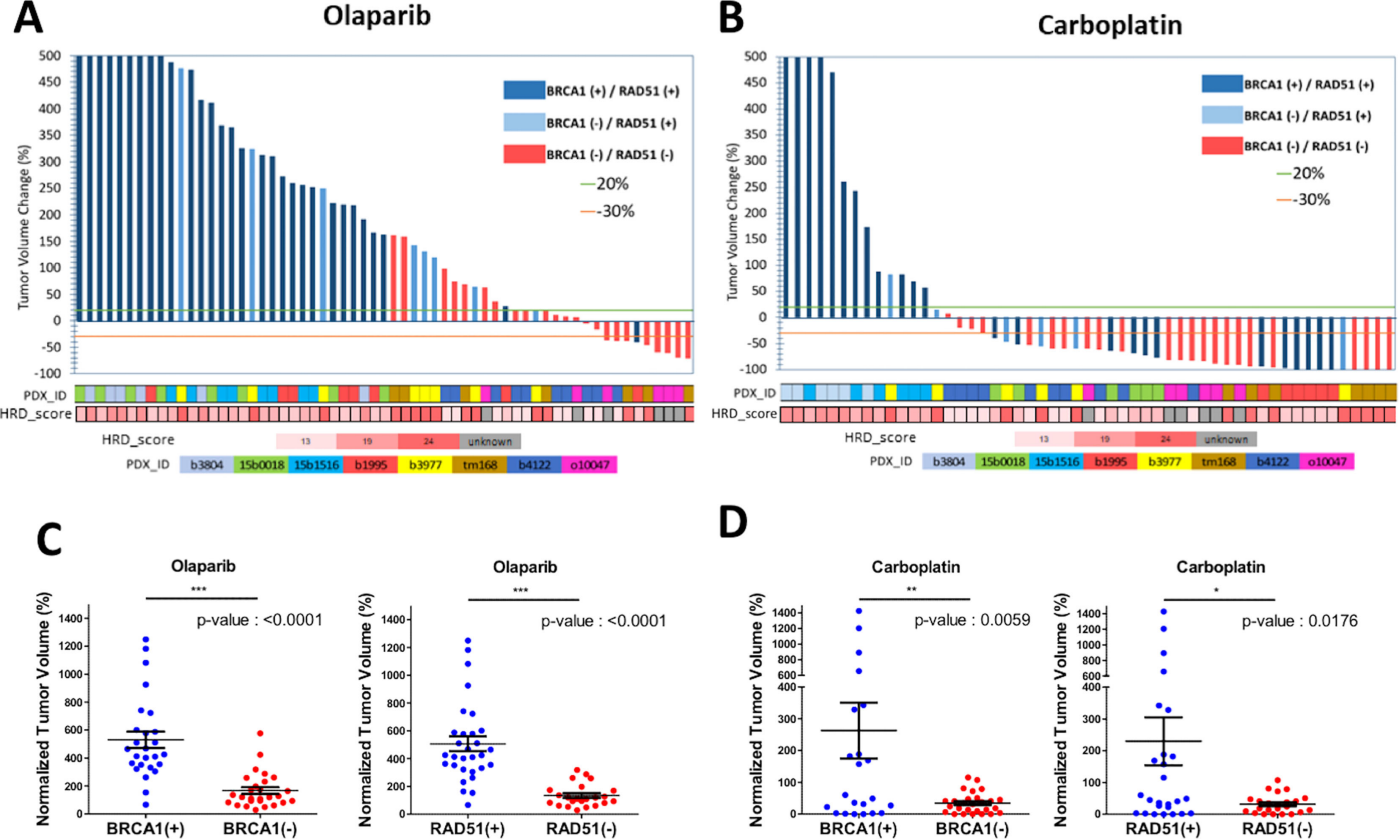
BRCA1 and RAD51 scores are good predictors of olaparib and Carboplatin response. Waterfall plots of the tumor volume change in individual PDX models (percentage of the starting volume) treated with either olaparib or Carboplatin. The status according to BRCA1 and/or RAD51 foci formation of each model is indicated by the color of the bar; dark blue BRCA1+/RAD51+; light blue BRCA1-/RAD51+; red BRCA1-/RAD51-. The identity of the PDX model is indicated by color boxes at the bottom of the graph. **(A)** olaparib treated mice. **(B)** Carboplatin treated mice. **(C)** tumor volume change under olaparib treatment stratified according to the BRCA1 foci status. **(D)** tumor volume change under olaparib treatment stratified according the RAD51 foci status. *, **,*** on top of the graph indicate the level of significance of the t-test.

As PDX responding to CBP represented twice the number of olaparib responders (6 *vs*. 3 respectively), we wanted to determine whether the response to CBP was associated with reduced RAD51 and/or BRCA1-foci formation (**Figures 1B, C)**. Like in the olaparib arm, individual grafts that responded to CBP were predominantly BRCA1-foci-negative/RAD51-foci-negative (**Figures 6A, B**) and, interestingly, most grafts of the BRCA-foci-negative/RAD51-foci-positive model b3977, whose response to olaparib was mediocre, showed tumor size reduction under CBP. However, grafts of the BRCA1-positive/RAD51-positive PDX 15b0018 and b1995, respectively *BRCA1*-*WT* and *BRCA1*-*Me* and bad responders to olaparib, showed good response to CBP pointing to the fact that CBP efficacy is not solely based on the HR status, but can also rely on alternative DNA repair mechanisms (**Figure 6B**). Despite these two BRCA1-foci-positive/RAD51-foci-positive models, both BRCA1-foci-negativity and RAD51-foci-negativity were significantly associated (t-test p-value 0.0059 and 0.0176 respectively) with CBP response in our dataset (**Figure 6D**). Next, we computed the Sensitivity, Specificity and Accuracy of RAD51, BRCA1, 53BP1 and γH2AX foci in predicting the response to olaparib and CBP (**Table 2**). Concerning olaparib response RAD51 foci showed high sensitivity (88%), specificity (82%) and accuracy (84%), while BRCA1 foci showed excellent sensitivity (94%) but lower specificity (66%) and accuracy (74%). Interestingly, when CBP response was considered, BRCA1 foci performed globally better showing 70% sensitivity, 92% Specificity and 75% accuracy in comparison with RAD51 foci, which showed 57% sensitivity, 100% Specificity but 68% accuracy. The performances of 53BP1 and γH2AX foci were overall rather contrasted showing excellent sensitivity (100%), but poor specificity (18%) and accuracy (41%) for olaparib response, while they reached better values for CBP response with high accuracy (87%) mitigating the mediocre specificity (46%). Overall, these data support the use of RAD51 foci for olaparib response prediction, while BRCA1 foci, possibly in combination with RAD51 foci, appear interesting for CBP response. While γH2AX and 53BP1 foci do not appear as convincing it may be interesting to reevaluate their performance for CBP response on a larger dataset.

**Table 2:** Test performance values for the indicated HRD biomarkers to predict olaparib and CBP response.

	Biomarkers	RAD51 Foci (n=61)	BRCA1 Foci (n=61)	γH2AX Foci (n=61)	53BP1 Foci (n=61)
**Olaparib response**	Sensitivity	88%	94%	100%	100%
	Specificity	82%	66%	18%	18%
	PPV	65%	52%	32%	32%
	NPV	95%	97%	100%	100%
	Accuracy	84%	74%	41%	41%
		(n=53)	(n=53)	(n=53)	(n=53)
**CBP response**	Sensitivity	57%	70%	100%	100%
	Specificity	100%	92%	46%	46%
	PPV	100%	97%	85%	85%
	NPV	43%	50%	100%	100%
	Accuracy	68%	75%	87%	87%

Response includes disease stabilization.

## Discussion

About one TNBC in three is estimated to be HR deficient. HRD has been linked with genetic or epigenetic impairment of genes belonging to the BRCA-pathway such as *BRCA1*, *BRCA2*, *PALB2* and *RAD51B*, *RAD51C* and *RAD51D*, but *BRCA1* is the most frequently affected gene in TNBC (5, 6). Because HR deficient tumors show increased sensitivity to PARPi or Platinum salts, detection of HRD has important implications in treatment definition (59). Identification of HRD is generally based on targeted sequencing of commonly mutated HR genes, combined with the determination of patterns of genomic rearrangements typical of HRD, such as the genomic HRD score, Tandem Duplication score or CX scores (20, 27, 31). However, BRCA-deficient cancers frequently develop resistance to treatment associated with HR restoration. Involved molecular events range from reverting secondary mutations, gene rearrangements producing gene chimeras, loss or mutations of the *53BP1* gene or of one of its cofactors in the Shieldin complex (34, 35, 59). Hence, the actual status of residual HRD tumors that have previously been exposed to genotoxic treatment could be in question. This point has been specifically raised concerning TNBC with silenced *BRCA1* gene due to hypermethylation of the promoter, whose sensitivity to PARPi has been disputed (31, 34).

These questions motivated the present study, where we interrogated the sensitivity to olaparib and CBP of 4 TNBC PDX models with epigenetically silenced *BRCA1*, 3 of which had received NACT prior PDX establishment. Noticeably, despite obliterated or severely reduced BRCA1 protein expression levels, the 3 *BRCA1*-methylated PDX that had undergone NACT responded poorly to olaparib, showing response profiles similar to those of the two *BRCA1-WT* models used as controls. These observations, thus, questioned the actual functionality of HR in the *BRCA1-Me* PDX models included in our study, leading us to test for BRCA1 and RAD51 nuclear foci formation in tissue sections of olaparib treated PDX and use this assay as a functional read out. In HR proficient cells, the BRCA1 and RAD51 proteins cluster onto DNA lesions and these clusters can be detected as nuclear foci by immunofluorescence microscopy (60). Their presence in tumor tissues signs for HR proficiency (61). Remarkably, the three *BRCA1-Me* PDX that responded poorly to olaparib scored positive for BRCA1 and/or RAD51 foci, likewise the two *BRCA1-WT* models. By contrast, the three PDX models responding to olaparib (2 *BRCA1-Mut* and 1 *BRCA1-Me*) scored negative for BRCA1 and RAD51-foci. Noticeably, the BRCA1 and RAD51 foci negative *BRCA1-Me* model had never been exposed to chemotherapy. Thus, our data suggest that HR was, at least partially, functional in the *BRCA1-Me* PDXs established from residual TNBC previously exposed to NACT, contributing to their poor response to olaparib. These observations were consistent with the increased RAD51 foci formation and HR restoration we evidenced in *BRCA1*-deficient cell line models that we rendered olaparib-resistant. Results presented herein support the notion that the actual HRD status of *BRCA1-Me* TNBC may be in doubt, especially if they have been previously exposed to genotoxic treatment. Since we did not have access to tumor samples prior NACT, we cannot conclude on HR restoration due to treatment exposure, but our data are in line with previous reports (31, 35). This calls for the verification of the HRD status based on functional read outs such as BRCA1 and/or RAD51 foci formation (62). Indeed, genetic tests or genomic scores yield valuable information on the HRD status of a given tumor, however, they point to its natural history and may be misleading in terms of actual HR functionality. We wish, moreover, to point out that all the PDX models included in our study presented elevated HRD or genetic instability scores, irrespective of their mutational, *BRCA1* methylation status or sensitivity to olaparib. Genetic and genomic scores are being considered concurrently with RAD51 foci determination as biomarkers predictive of treatment in TNBC and prostate cancer (63–65). Results support the excellent correlation between low RAD51 scores and HR deficiency, as well as with increased sensitivity to olaparib or platinum based regimen (63, 65, 66). However, these works also point out the detection of tumors showing poor response to treatment, whilst presenting inactivating BRCA1 or BRCA2 mutations or elevated HRD scores and scoring positive for RAD51 foci. Our data are in perfect concordance with these observations and further support the relevance of BRCA1 and/or RAD51 foci-based tests to determine the functionality of HR in TNBC presenting all signs or HRD (63, 65, 66). Further, our data highlight the importance of verifying the actual functionality of HRR in tumors with epigenetically silenced *BRCA1*, particularly those previously exposed to chemotherapy during the course of the disease.

A number of studies highlight the exquisite sensitivity of HRD tumors to cis or carboplatin, alone or in combination with other molecules (63, 65, 66). We thus, documented the sensitivity to CBP of our models and determine their overlap with BRCA1 and RAD51 scores. PDX models responding to CBP were twice more frequent than olaparib responders and included all BRCA1-foci negative cases, as well as 2 BRCA1-foci/RAD51-foci positive PDX models, underlining the fact that CBP sensitivity is not solely determined by the HRD status. Indeed, platinum salts produces bulky adducts that must be removed by NER to avoid DNA breaks and tumors with faulty NER have been shown to be highly sensitive to platinum (59, 67, 68). Hence, while BRCA1 foci appeared a better predictor of CBP sensitivity than RAD51 in our dataset, they missed 2 out 6 CBP sensitive cases, thus calling for complementary tests.

## Conclusion

Our work shows that TNBC with a silenced *BRCA1* gene, due to hypermethylation of its promoter, may be prone to HR restoration and, thus, become resistant to olaparib. Interestingly, two of the olaparib resistant and RAD51-foci positive PDX models appeared sensitive to CBP. Our data thus support the notion that the HRD status of TNBC should be systematically checked using a combination of biomarkers, among which the BRCA1 and RAD51 foci formation tests play a major role. Not only do these assays inform on HR functionality in a given tumor, they are cheap, rapid and quite easy to implement. We performed the immunofluorescence analysis on olaparib treated tumor samples to ensure a clear signal difference between foci positive and foci negative samples, but noted, in accordance with other works, that the difference was also perceptible in non-treated tumors (63). Hence, this test, which we and others have shown to reliably predict sensitivity to olaparib, and also to CBP, could be implemented on Formalin fixed tumors as part of a pathology routine in a number of cancer treating institutions.

## Data availability statement

The raw data supporting the conclusions of this article will be made available by the authors, without undue reservation.

## Ethics statement

The animal study was reviewed and approved by CEEA-LR-12028.

## Author contributions

Conceptualization: CT. Funding acquisition: WJ and CT. Investigation: CV, EO, IT, LF, JA, and EC. Methodology and resources: BO, MC, and AG. Supervision: CT and WJ. Validation: DB, CS, WJ, and CT. Writing: CT and WJ. All authors contributed to the article and approved the submitted version.

## References

[1] Dent R Trudeau M Pritchard KI Hanna WM Kahn HK Sawka CA. Triple-negative breast cancer: clinical features and patterns of recurrence. Clin Cancer Res (2007) 13:4429–34. doi: 10.1158/1078-0432.CCR-06-3045 17671126

[2] Elias AD. Triple-negative breast cancer: A short review. Am J Clin Oncol (2010) 33:637–45. doi: 10.1097/COC.0b013e3181b8afcf 20023571

[3] Carey LA Dees EC Sawyer L Gatti L Moore DT Collichio F. The triple negative paradox: Primary tumor chemosensitivity of breast cancer subtypes. Clin Cancer Res (2007) 13:2329–34. doi: 10.1158/1078-0432.CCR-06-1109 17438091

[4] Zagami P Carey LA. Triple negative breast cancer: Pitfalls and progress. NPJ Breast Cancer. (2022) 8:95. doi: 10.1038/s41523-022-00468-0 35987766 PMC9392735

[5] Stephens PJ Tarpey PS Davies H Van Loo P Greenman C Wedge DC. The landscape of cancer genes and mutational processes in breast cancer. Nature. (2012) 486:400–4. doi: 10.1038/nature11017 PMC3428862 22722201

[6] Couch FJ Hart SN Sharma P Toland AE Wang X Miron P. Inherited mutations in 17 breast cancer susceptibility genes among a Large triple-negative breast cancer cohort unselected for family history of breast cancer. JCO. (2015) 33:304–11. doi: 10.1200/JCO.2014.57.1414 PMC4302212 25452441

[7] Lord CJ Ashworth A. BRCAness revisited. Nat Rev Cancer. (2016) 16:110–20. doi: 10.1038/nrc.2015.21 26775620

[8] Roy R Chun J Powell SN. BRCA1 and BRCA2: different roles in a common pathway of genome protection. Nat Rev Cancer. (2012) 12:68–78. doi: 10.1038/nrc3181 PMC4972490 22193408

[9] Helleday T Eshtad S Nik-Zainal S. Mechanisms underlying mutational signatures in human cancers. Nat Rev Genet (2014) 15:585–98. doi: 10.1038/nrg3729 PMC6044419 24981601

[10] Hastak K Alli E Ford JM. Synergistic chemosensitivity of triple-negative breast cancer cell lines to Poly(ADP-ribose) polymerase inhibition, gemcitabine, and cisplatin. Cancer Res (2010) 70:7970–80. doi: 10.1158/0008-5472.CAN-09-4521 PMC2955854 20798217

[11] Silver DP Richardson AL Eklund AC Wang ZC Szallasi Z Li Q. Efficacy of neoadjuvant cisplatin in triple-negative breast cancer. J Clin Oncol (2010) 28:1145–53. doi: 10.1200/JCO.2009.22.4725 PMC2834466 20100965

[12] Sikov WM Berry DA Perou CM Singh B Cirrincione CT Tolaney SM. Impact of the addition of carboplatin and/or bevacizumab to neoadjuvant once-per-week paclitaxel followed by dose-dense doxorubicin and cyclophosphamide on pathologic complete response rates in stage II to III triple-negative breast cancer: CALGB 40603 (Alliance). J Clin Oncol (2015) 33:13–21. doi: 10.1200/JCO.2014.57.0572 25092775 PMC4268249

[13] Fong PC Boss DS Yap TA Tutt A Wu P Mergui-Roelvink M. Inhibition of poly(ADP-ribose) polymerase in tumors from BRCA mutation carriers. N Engl J Med (2009) 361:123–34. doi: 10.1056/NEJMoa0900212 19553641

[14] Tutt A Robson M Garber JE Domchek SM Audeh MW Weitzel JN. Oral poly(ADP-ribose) polymerase inhibitor olaparib in patients with BRCA1 or BRCA2 mutations and advanced breast cancer: a proof-of-concept trial. Lancet (2010) 376:235–44. doi: 10.1016/S0140-6736(10)60892-6 20609467

[15] Audeh MW Carmichael J Penson RT Friedlander M Powell B Bell-McGuinn KM. Oral poly(ADP-ribose) polymerase inhibitor olaparib in patients with BRCA1 or BRCA2 mutations and recurrent ovarian cancer: a proof-of-concept trial. Lancet (2010) 376:245–51. doi: 10.1016/S0140-6736(10)60893-8 20609468

[16] Robson M Im S-A Senkus E Xu B Domchek SM Masuda N. Olaparib for metastatic breast cancer in patients with a germline *BRCA* mutation. N Engl J Med (2017) 377:523–33. doi: 10.1056/NEJMoa1706450 28578601

[17] Davies H Glodzik D Morganella S Yates LR Staaf J Zou X. HRDetect is a predictor of BRCA1 and BRCA2 deficiency based on mutational signatures. Nat Med (2017) 23:517–25. doi: 10.1038/nm.4292 PMC5833945 28288110

[18] Polak P Kim J Braunstein LZ Karlic R Haradhavala NJ Tiao G. A mutational signature reveals alterations underlying deficient homologous recombination repair in breast cancer. Nat Genet (2017) 49:1476–86. doi: 10.1038/ng.3934 PMC7376751 28825726

[19] Menghi F Barthel FP Yadav V Tang M Ji B Tang Z. The tandem duplicator phenotype is a prevalent genome-wide cancer configuration driven by distinct gene mutations. Cancer Cell (2018) 34:197–210.e5. doi: 10.1016/j.ccell.2018.06.008 30017478 PMC6481635

[20] Drews RM Hernando B Tarabichi M Haase K Lesluyes T Smith PS. A pan-cancer compendium of chromosomal instability. Nature. (2022) 606:976–83. doi: 10.1038/s41586-022-04789-9 PMC7613102 35705807

[21] Glodzik D Bosch A Hartman J Aine M Vallon-Christersson J Reuterswärd C. Comprehensive molecular comparison of BRCA1 hypermethylated and BRCA1 mutated triple negative breast cancers. Nat Commun (2020) 11:3747. doi: 10.1038/s41467-020-18098-0 32719340 PMC7385112

[22] Pujol P Yauy K Coffy A Duforet-Frebourg N Gabteni S Daurès J-P. Predominance of BRCA2 mutation and estrogen receptor positivity in unselected breast cancer with BRCA1 or BRCA2 mutation. Cancers. (2022) 14:3266. doi: 10.3390/cancers14133266 35805038 PMC9265086

[23] Armstrong N Ryder S Forbes C Ross J Quek RG. A systematic review of the international prevalence of BRCA mutation in breast cancer. CLEP (2019) 11:543–61. doi: 10.2147/CLEP.S206949 PMC6628947 31372057

[24] Telli ML Timms KM Reid J Hennessy B Mills GB Jensen KC. Homologous recombination deficiency (HRD) score predicts response to platinum-containing neoadjuvant chemotherapy in patients with triple-negative breast cancer. Clin Cancer Res (2016) 22:3764–73. doi: 10.1158/1078-0432.CCR-15-2477 PMC6773427 26957554

[25] Tutt A Tovey H Cheang MCU Kernaghan S Kilburn L Gazinska P. Carboplatin in BRCA1/2-mutated and triple-negative breast cancer BRCAness subgroups: the TNT trial. Nat Med (2018) 24:628–37. doi: 10.1038/s41591-018-0009-7 PMC6372067 29713086

[26] Staaf J Glodzik D Bosch A Vallon-Christersson J Reuterswärd C Häkkinen J. Whole-genome sequencing of triple-negative breast cancers in a population-based clinical study. Nat Med (2019) 25:1526–33. doi: 10.1038/s41591-019-0582-4 PMC6859071 31570822

[27] Chopra N Tovey H Pearson A Cutts R Toms C Proszek P. Homologous recombination DNA repair deficiency and PARP inhibition activity in primary triple negative breast cancer. Nat Commun (2020) 11:2662. doi: 10.1038/s41467-020-16142-7 32471999 PMC7260192

[28] Jacot W Lopez-Crapez E Mollevi C Boissière-Michot F Simony-Lafontaine J Ho-Pun-Cheung A. BRCA1 promoter hypermethylation is associated with good prognosis and chemosensitivity in triple-negative breast cancer. Cancers. (2020) 12:828. doi: 10.3390/cancers12040828 32235500 PMC7225997

[29] Drew Y Mulligan EA Vong W-T Thomas HD Kahn S Kyle S. Therapeutic potential of poly(ADP-ribose) polymerase inhibitor AG014699 in human cancers with mutated or methylated BRCA1 or BRCA2. J Natl Cancer Inst (2011) 103:334–46. doi: 10.1093/jnci/djq509 21183737

[30] Veeck J Ropero S Setien F Gonzalez-Suarez E Osorio A Benitez J. BRCA1 CpG island hypermethylation predicts sensitivity to poly(adenosine diphosphate)-ribose polymerase inhibitors. J Clin Oncol (2010) 28:e563–564. doi: 10.1200/JCO.2010.30.1010 20679605

[31] Menghi F Banda K Kumar P Straub R Dobrolecki L Rodriguez IV. Genomic and epigenomic BRCA alterations predict adaptive resistance and response to platinum-based therapy in patients with triple-negative breast and ovarian carcinomas. Sci Transl Med (2022) 14:eabn1926. doi: 10.1126/scitranslmed.abn1926 35857626 PMC9585706

[32] Eikesdal HP Yndestad S Elzawahry A Llop-Guevara A Gilje B Blix ES. Olaparib monotherapy as primary treatment in unselected triple negative breast cancer. Ann Oncol (2021) 32:240–9. doi: 10.1016/j.annonc.2020.11.009 33242536

[33] D’Andrea AD. Mechanisms of PARP inhibitor sensitivity and resistance. DNA Repair (2018) 71:172–6. doi: 10.1016/j.dnarep.2018.08.021 30177437

[34] Dias MP Moser SC Ganesan S Jonkers J. Understanding and overcoming resistance to PARP inhibitors in cancer therapy. Nat Rev Clin Oncol (2021) 18:773–91. doi: 10.1038/s41571-021-00532-x 34285417

[35] Ter Brugge P Kristel P van der Burg E Boon U de Maaker M Lips E. Mechanisms of therapy resistance in patient-derived xenograft models of BRCA1-deficient breast cancer. J Natl Cancer Inst (2016) 108:1–12. doi: 10.1093/jnci/djw148 27381626

[36] du Manoir S Orsetti B Bras-Gonçalves R Nguyen T-T Lasorsa L Boissière F. Breast tumor PDXs are genetically plastic and correspond to a subset of aggressive cancers prone to relapse. Mol Oncol (2014) 8:431–43. doi: 10.1016/j.molonc.2013.11.010 PMC5528550 24394560

[37] Colombo P-E du Manoir S Orsetti B Bras-Gonçalves R Lambros MB MacKay A. Ovarian carcinoma patient derived xenografts reproduce their tumor of origin and preserve an oligoclonal structure. Oncotarget. (2015) 6:28327–40. doi: 10.18632/oncotarget.5069 PMC4695063 26334103

[38] Adélaïde J Finetti P Bekhouche I Repellini L Geneix J Sircoulomb F. Integrated profiling of basal and luminal breast cancers. Cancer Res (2007) 67:11565–75. doi: 10.1158/0008-5472.CAN-07-2536 18089785

[39] Abkevich V Timms KM Hennessy BT Potter J Carey MS Meyer LA. Patterns of genomic loss of heterozygosity predict homologous recombination repair defects in epithelial ovarian cancer. Br J Cancer. (2012) 107:1776–82. doi: 10.1038/bjc.2012.451 PMC3493866 23047548

[40] Bertucci F Finetti P Guille A Adélaïde J Garnier S Carbuccia N. Comparative genomic analysis of primary tumors and metastases in breast cancer. Oncotarget. (2016) 7:27208–19. doi: 10.18632/oncotarget.8349 PMC5053643 27028851

[41] Li H Durbin R. Fast and accurate short read alignment with burrows-wheeler transform. Bioinformatics. (2009) 25:1754–60. doi: 10.1093/bioinformatics/btp324 PMC2705234 19451168

[42] Garrison E Marth G. Haplotype-based variant detection from short-read sequencing (2012). Available at: https://arxiv.org/abs/1207.3907.

[43] Poplin R Ruano-Rubio V DePristo MA Fennell TJ Carneiro MO van der Auwera GA. Scaling accurate genetic variant discovery to tens of thousands of samples. bioRxiv (2017). doi: 10.1101/201178

[44] Wilm A Aw PPK Bertrand D Yeo GHT Ong SH Wong CH. LoFreq: a sequence-quality aware, ultra-sensitive variant caller for uncovering cell-population heterogeneity from high-throughput sequencing datasets. Nucleic Acids Res (2012) 40:11189–201. doi: 10.1093/nar/gks918 PMC3526318 23066108

[45] Auwera GAVd O’Connor BD. Genomics in the cloud: Using docker, GATK, and WDL in Terra. O’Reilly Media (2020).

[46] Dunn T Berry G Emig-Agius D Jiang Y Lei S Iyer A. Pisces: an accurate and versatile variant caller for somatic and germline next-generation sequencing data. Bioinformatics (2019) 35:1579–81. doi: 10.1093/bioinformatics/bty849 PMC6499249 30304370

[47] Rimmer A Phan H Mathieson I Iqbal Z Twigg SRF Wilkie AOM. Integrating mapping-, assembly- and haplotype-based approaches for calling variants in clinical sequencing applications. Nat Genet (2014) 46:912–8. doi: 10.1038/ng.3036 PMC4753679 25017105

[48] Lai Z Markovets A Ahdesmaki M Chapman B Hofmann O McEwen R. VarDict: a novel and versatile variant caller for next-generation sequencing in cancer research. Nucleic Acids Res (2016) 44:e108–8. doi: 10.1093/nar/gkw227 PMC4914105 27060149

[49] Koboldt DC Zhang Q Larson DE Shen D McLellan MD Lin L. VarScan 2: Somatic mutation and copy number alteration discovery in cancer by exome sequencing. Genome Res (2012) 22:568–76. doi: 10.1101/gr.129684.111 PMC3290792 22300766

[50] Ye K Schulz MH Long Q Apweiler R Ning Z. Pindel: a pattern growth approach to detect break points of large deletions and medium sized insertions from paired-end short reads. Bioinformatics. (2009) 25:2865–71. doi: 10.1093/bioinformatics/btp394 PMC2781750 19561018

[51] Fang H Bergmann EA Arora K Vacic V Zody MC Iossifov I. Indel variant analysis of short-read sequencing data with scalpel. Nat Protoc (2016) 11:2529–48. doi: 10.1038/nprot.2016.150 PMC5507611 27854363

[52] Robinson JT Thorvaldsdóttir H Winckler W Guttman M Lander ES Getz G. Integrative genomics viewer. Nat Biotechnol (2011) 29:24–6. doi: 10.1038/nbt.1754 PMC3346182 21221095

[53] Thorvaldsdottir H Robinson JT Mesirov JP. Integrative genomics viewer (IGV): high-performance genomics data visualization and exploration. Briefings Bioinf (2013) 14:178–92. doi: 10.1093/bib/bbs017 PMC3603213 22517427

[54] Bertucci F Rypens C Finetti P Guille A Adélaïde J Monneur A. NOTCH and DNA repair pathways are more frequently targeted by genomic alterations in inflammatory than in non-inflammatory breast cancers. Mol Oncol (2020) 14:504–19. doi: 10.1002/1878-0261.12621 PMC7053236 31854063

[55] Manoir S Delpech H Orsetti B Jacot W Pirot N Noel J. In high-grade ovarian carcinoma, platinum-sensitive tumor recurrence and acquired-resistance derive from quiescent residual cancer cells that overexpress CRYAB , CEACAM6, and SOX2 . J Pathology. (2022) 257:367–78. doi: 10.1002/path.5896 35302657

[56] Wang Y Bernhardy AJ Cruz C Krais JJ Nacson J Nicolas E. The BRCA1-Δ11q alternative splice isoform bypasses germline mutations and promotes therapeutic resistance to PARP inhibition and cisplatin. Cancer Res (2016) 76:2778–90. doi: 10.1158/0008-5472.CAN-16-0186 PMC4874568 27197267

[57] Elstrodt F Hollestelle A Nagel JHA Gorin M Wasielewski M van den Ouweland A. *BRCA1* mutation analysis of 41 human breast cancer cell lines reveals three new deleterious mutants. Cancer Res (2006) 66:41–5. doi: 10.1158/0008-5472.CAN-05-2853 16397213

[58] DelloRusso C Welcsh PL Wang W Garcia RL King M-C Swisher EM. Functional characterization of a novel BRCA1-null ovarian cancer cell line in response to ionizing radiation. Mol Cancer Res (2007) 5:35–45. doi: 10.1158/1541-7786.MCR-06-0234 17259345

[59] Konstantinopoulos PA Ceccaldi R Shapiro GI D’Andrea AD. Homologous recombination deficiency: Exploiting the fundamental vulnerability of ovarian cancer. Cancer Discovery. (2015) 5:1137–54. doi: 10.1158/2159-8290.CD-15-0714 PMC4631624 26463832

[60] Willers H Taghian AG Luo C-M Treszezamsky A Sgroi DC Powell SN. Utility of DNA repair protein foci for the detection of putative BRCA1 pathway defects in breast cancer biopsies. Mol Cancer Res (2009) 7:1304–9. doi: 10.1158/1541-7786.MCR-09-0149 PMC4239295 19671671

[61] Graeser M McCarthy A Lord CJ Savage K Hills M Salter J. A marker of homologous recombination predicts pathologic complete response to neoadjuvant chemotherapy in primary breast cancer. Clin Cancer Res (2010) 16:6159–68. doi: 10.1158/1078-0432.CCR-10-1027 PMC3432445 20802015

[62] van Wijk LM Kramer CJH Vermeulen S ter Haar NT de Jonge MM Kroep JR. The RAD51-FFPE test; calibration of a functional homologous recombination deficiency test on diagnostic endometrial and ovarian tumor blocks. Cancers. (2021) 13:2994. doi: 10.3390/cancers13122994 34203855 PMC8232577

[63] Castroviejo-Bermejo M Cruz C Llop-Guevara A Gutiérrez-Enríquez S Ducy M Ibrahim YH. A RAD 51 assay feasible in routine tumor samples calls PARP inhibitor response beyond BRCA mutation. EMBO Mol Med (2018) 10:1–16. doi: 10.15252/emmm.201809172 PMC6284440 30377213

[64] Carreira S Porta N Arce-Gallego S Seed G Llop-Guevara A Bianchini D. Biomarkers associating with PARP inhibitor benefit in prostate cancer in the TOPARP-b trial. Cancer Discovery. (2021) 11:2812–27. doi: 10.1158/2159-8290.CD-21-0007 PMC9414325 34045297

[65] Pellegrino B Herencia-Ropero A Llop-Guevara A Pedretti F Moles-Fernández A Viaplana C. Preclinical *In vivo* validation of the RAD51 test for identification of homologous recombination-deficient tumors and patient stratification. Cancer Res (2022) 82:1646–57. doi: 10.1158/0008-5472.CAN-21-2409 PMC7612637 35425960

[66] Llop-Guevara A Loibl S Villacampa G Vladimirova V Schneeweiss A Karn T. Association of RAD51 with homologous recombination deficiency (HRD) and clinical outcomes in untreated triple-negative breast cancer (TNBC): analysis of the GeparSixto randomized clinical trial. Ann Oncol (2021) 32:1590–6. doi: 10.1016/j.annonc.2021.09.003 34520831

[67] Reardon JT Vaisman A Chaney SG Sancar A. Efficient nucleotide excision repair of cisplatin, oxaliplatin, and bis-aceto-ammine-dichloro-cyclohexylamine-platinum(IV) (JM216) platinum intrastrand DNA diadducts. Cancer Res (1999) 59:3968–71. 10463593

[68] Ceccaldi R O’Connor KW Mouw KW Li AY Matulonis UA D’Andrea AD. A unique subset of epithelial ovarian cancers with platinum sensitivity and PARP inhibitor resistance. Cancer Res (2015) 75:628–34. doi: 10.1158/0008-5472.CAN-14-2593 PMC4416405 25634215

