# Supplementary material for: BRCA1-methylated triple negative breast cancers previously exposed to neoadjuvant chemotherapy form RAD51 foci and respond poorly to olaparib
Source: Front Oncol. 2023 Mar 17;13:1125021. doi: 10.3389/fonc.2023.1125021 (PMC10064050; doi:10.3389/fonc.2023.1125021)
Supplement: Supplementary file 1 [file DataSheet_1.pdf]

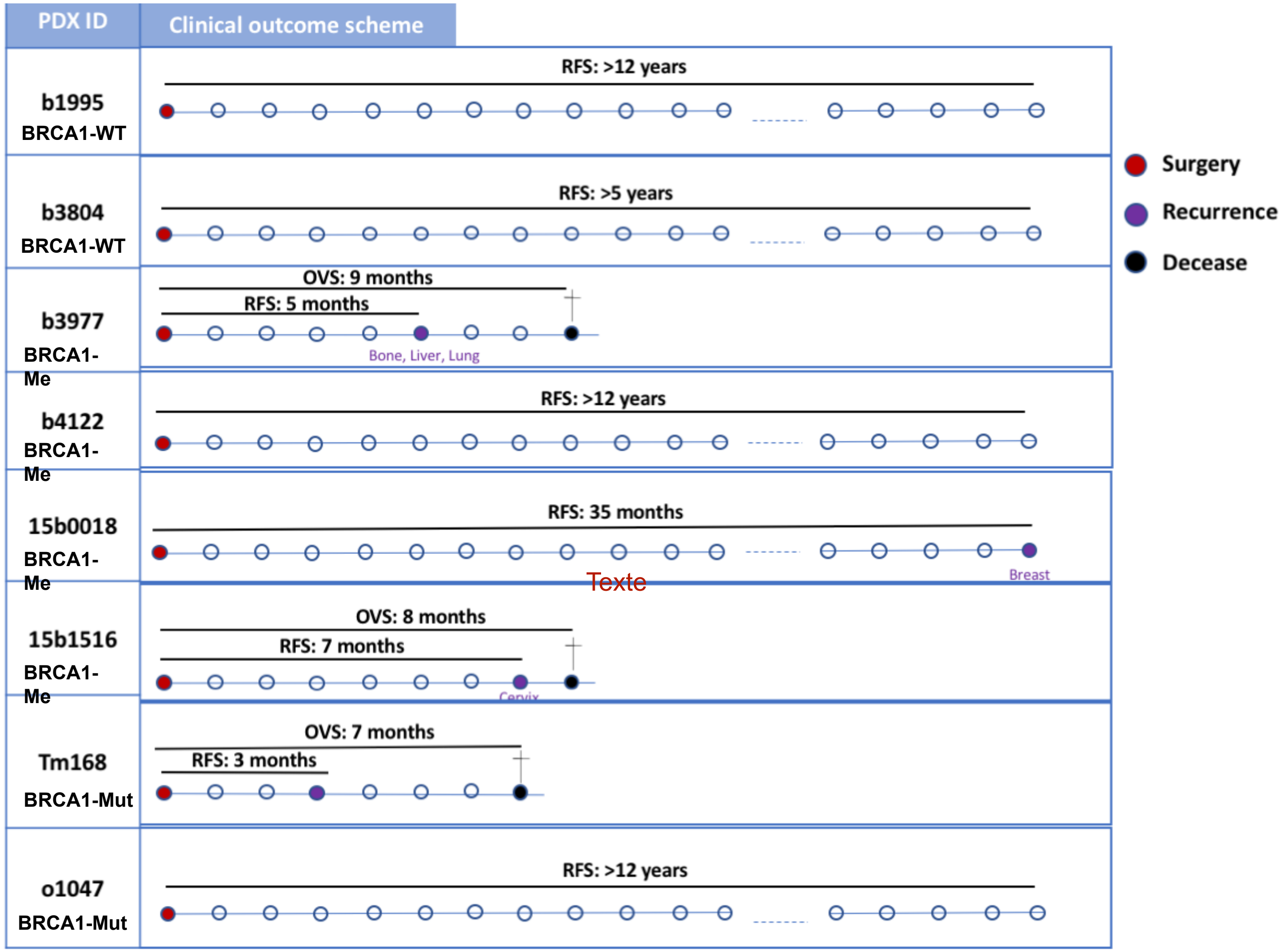

Supplementary Figure 1: patient follow up data of the original tumors from which the PDX have been derived

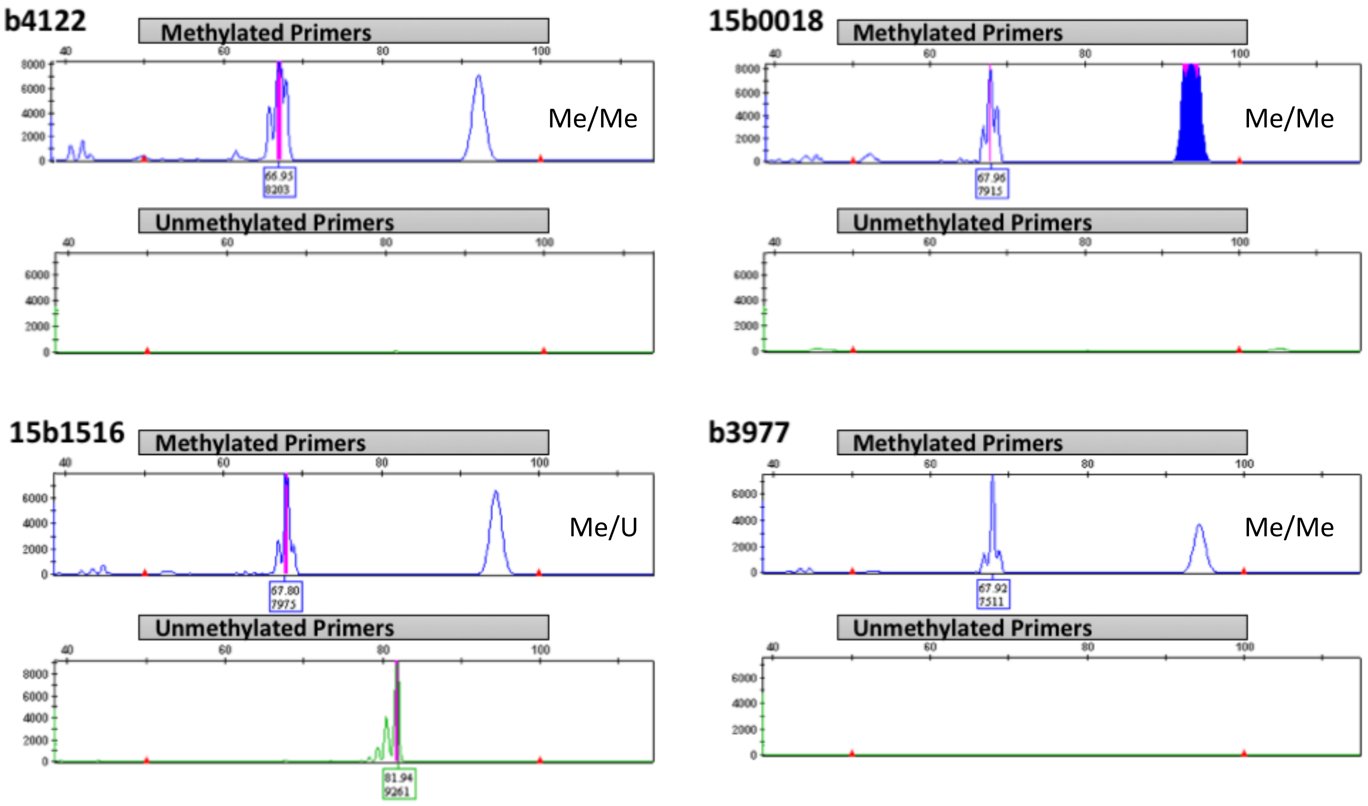

Supplementary Figure 2: Methylation Specific (MS-PCR) analysis of the 4 PDX with BRCA1 promoter hypermethylation.

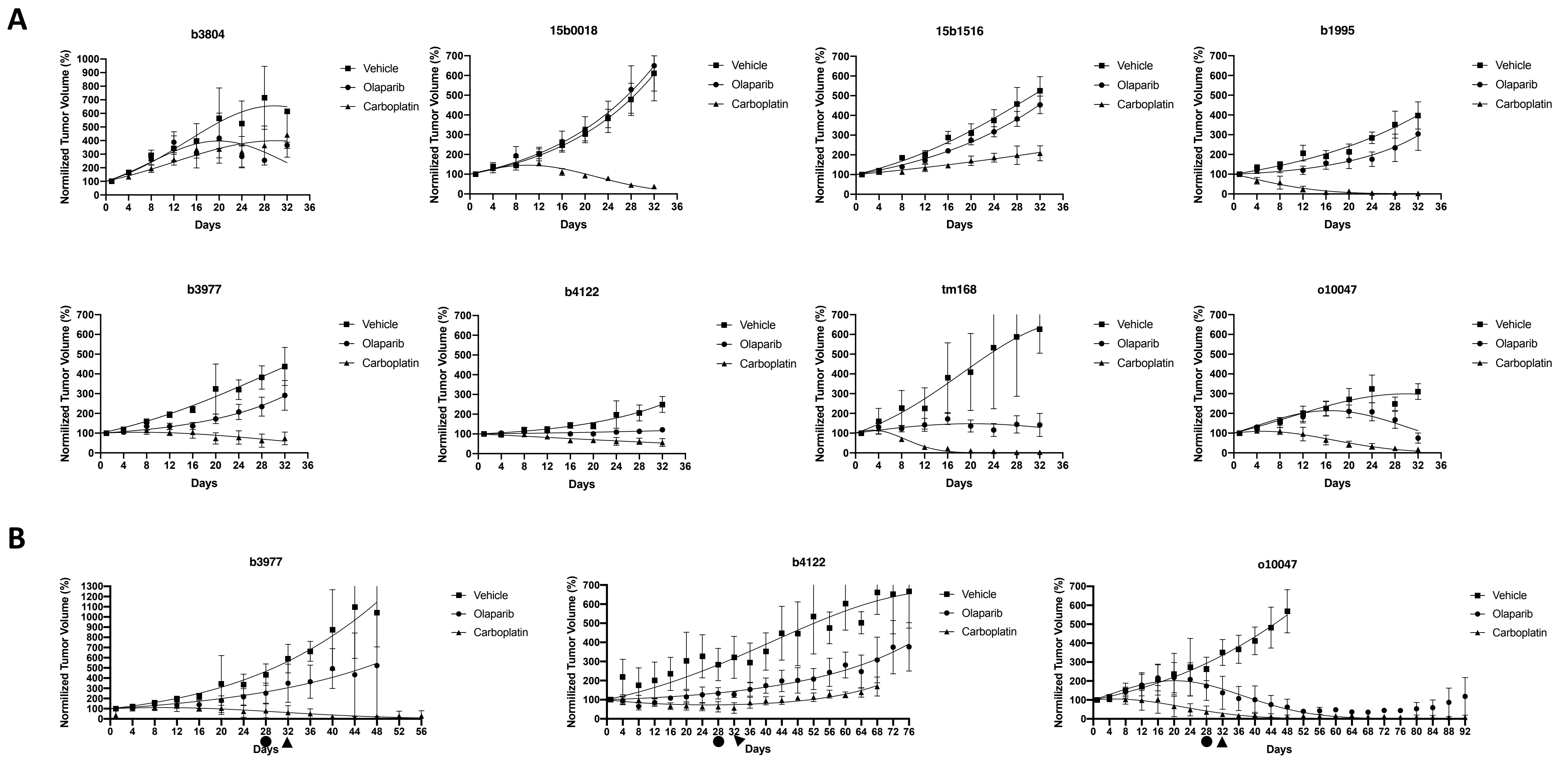

Supplementary Figure 3: Response to Olaparib and Carboplatin of the 8 PDX models. **A**; tumor growth curves in each experimental arm (vehicle, Olaparib, Carboplatin) were expressed as the mean of normalized tumor volume (TV). Carboplatin was administrated intraperitoneally at 50 mg/kg twice a week for 4 weeks. Olaparib was administrated by oral gavage at 100 mg/kg 5days/week for 5 weeks. **B**; PDX b3977, b4122 and 010047 were monitorited after end of treatment for tumor regrowth. Black circles and arrowhead indicate end of CBP and olaparib treatment respectively.

	b3804 WT	b1995 WT	15b0018 Me/Me	15b1516 Me/UM	b3977 Me/Me	b4122 Me/Me	tm168 Mut	o10047 Mut
BRCA1	0	0	-0.65	-0.5	-0.5	-0.85	0	0
BRCA2	0	-0.8	-0.4	0	-0.5	0	0	0
PALB2	0	-0.8	0	0	-0.6	0	0	-0.5
RAD51	0	0	-0.6	-0.4	-0.6	-0.9	0.7	-0.9
RAD51B	0	-0.4	-0.5	0	-0.5	-0.6	0	0.95
RAD51C	0	0	-0.6	-0.55	0	-0.3	0.6	-0.45
RAD51D	0	-0.7	0	-0.5	0.6	0	-0.3	-0.92
CDK12	0	0	0	-0.5	-0.6	-0.85	0	0.72
PARP1	0	0.7	0	0	0	0	0.8	0
53BP1	0	0	-0.7	-0.4	-0.6	-0.9	0	0
RIF1	0	0	0	0	0	0	-0.3	0.8
MAD2L2/REV7	0	0	-0.7	-1.1	0	-0.95	0	0
ATM	0	0	0	-0.5	-0.7	0	-0.4	0
ATR	0	0	0	0	0	0	0.6	0.6
PTEN	0	0	-1.3	0	-0.5	1	-0.4	0
ERCC2	-0.5	0	-0.7	0	0	0	0	-0.5
ERCC3	0	0.6	0	0	0	0	-0.3	0.55
ERCC4	0	-0.8	0	0	-0.6	0	0	0
ERCC5	0.6	0	-0.4	0	-0.4	0	0.9	0
SETD2	0	-0.9	-0.4	-0.5	-0.7	-0.9	0	-1.3
ARID1A	0	0	0	-0.4	-0.7	-0.9	0	0
ABCB1	-0.3	0	-0.35	0	-0.35	0	0	0
STK11/LKB1	0	-0.9	-1.5	-0.5	-0.65	-0.5	0.5	-1.11
MYC	1	0	1	1.5	2	1.3	1.6	1.34
KRAS	1.9	0	-0.3	0.6	0	0.8	0	1.16
CCND1	1.3	0	0	-0.3	-0.5	0	0	0.54
CCND2	1.4	0	0	0	0.8	0.9	0	0.8
CCNE1	-0.4	0	1	0	0	-0.8	0.6	-0.4

-1.5	-1.0	-0.5	0	0.5	1.0	1.5	2.0	**Log2 ratio**
0.7	1.0	1.4	2	3	4	6	8	**Copy Number**

Supplementary Figure 4: copy number changes and chromosomal instability scores of the tested TNBC PDX models. **A**: copy number changes involving genes in the HR and NER pathways. Copy numbers determined using CGH-array Agilent arrays and expressed as Log2 of the fluorescence ratio of the Normal reference DNA and tumor DNA. Copy Numbers were infered using a diploid genome as reference.

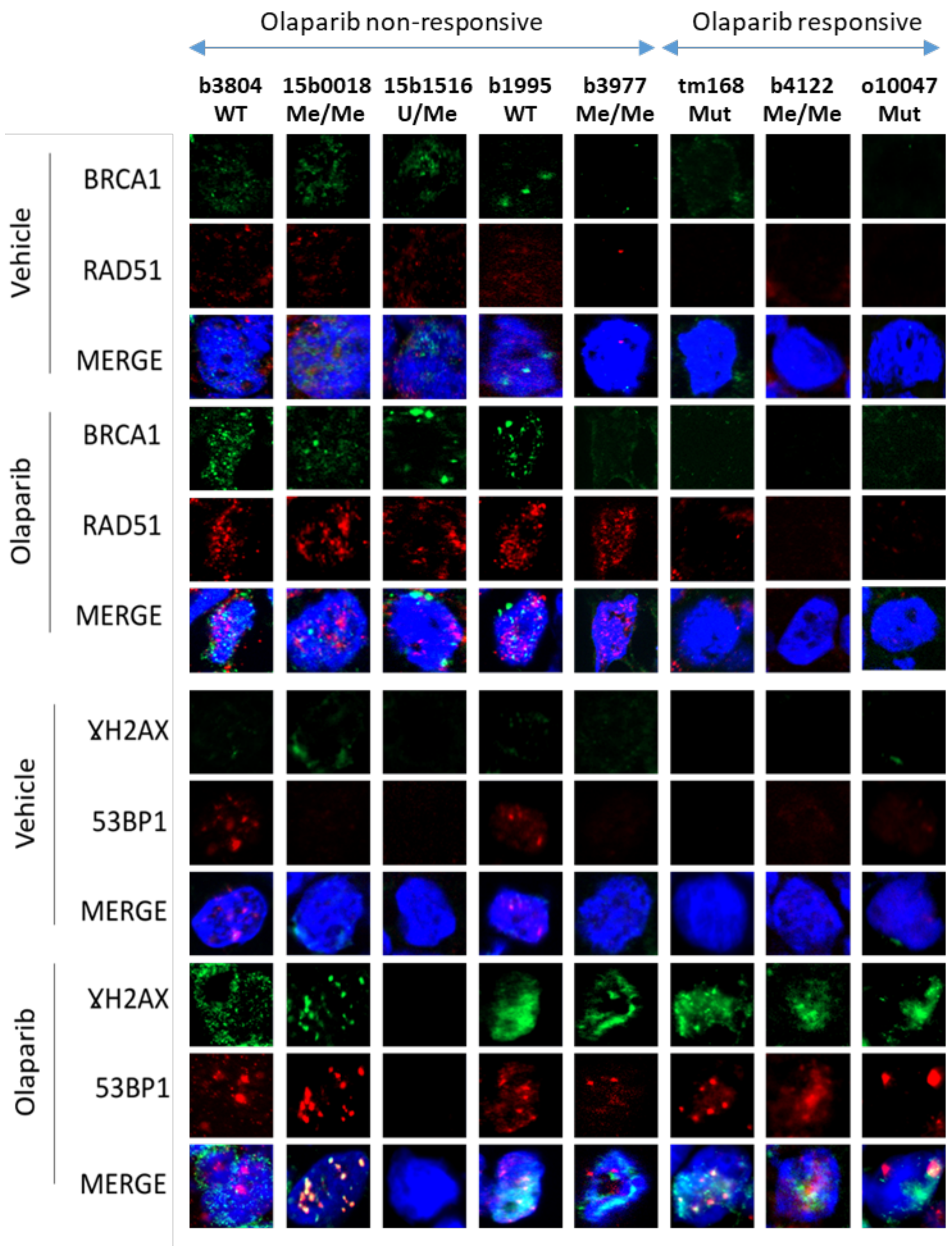

Supplementary Figure 5: BRCA1, RAD51, γH2AX and 53BP1 nuclear foci in olaparib-treated PDX models. Data complementing results in Figure 3A

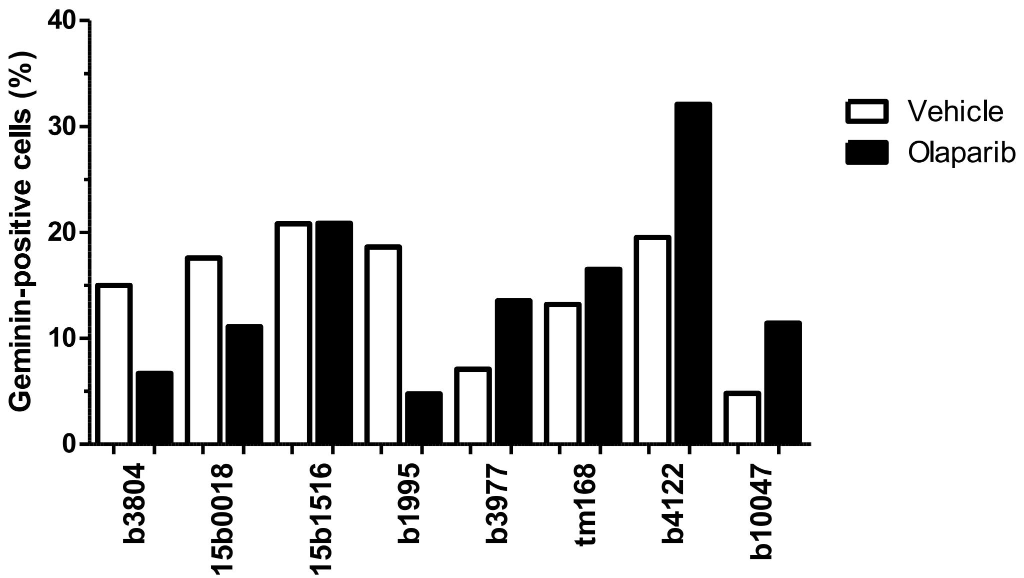

Supplementary Figure 6: **Quantification of Geminin positive cells in vehicle and Olaparib treated PDXs**. Frozen tissue sections from vehicle and olaparib treated PDX were sampled as described in Figure 3 and the fraction (%) of cells showing positive staining for Geminin a protein associated to DNA replication determined by immunofulorescence. Note that the fraction Geminin-positive cells remained stable or increased in most olaparib treated PDX.

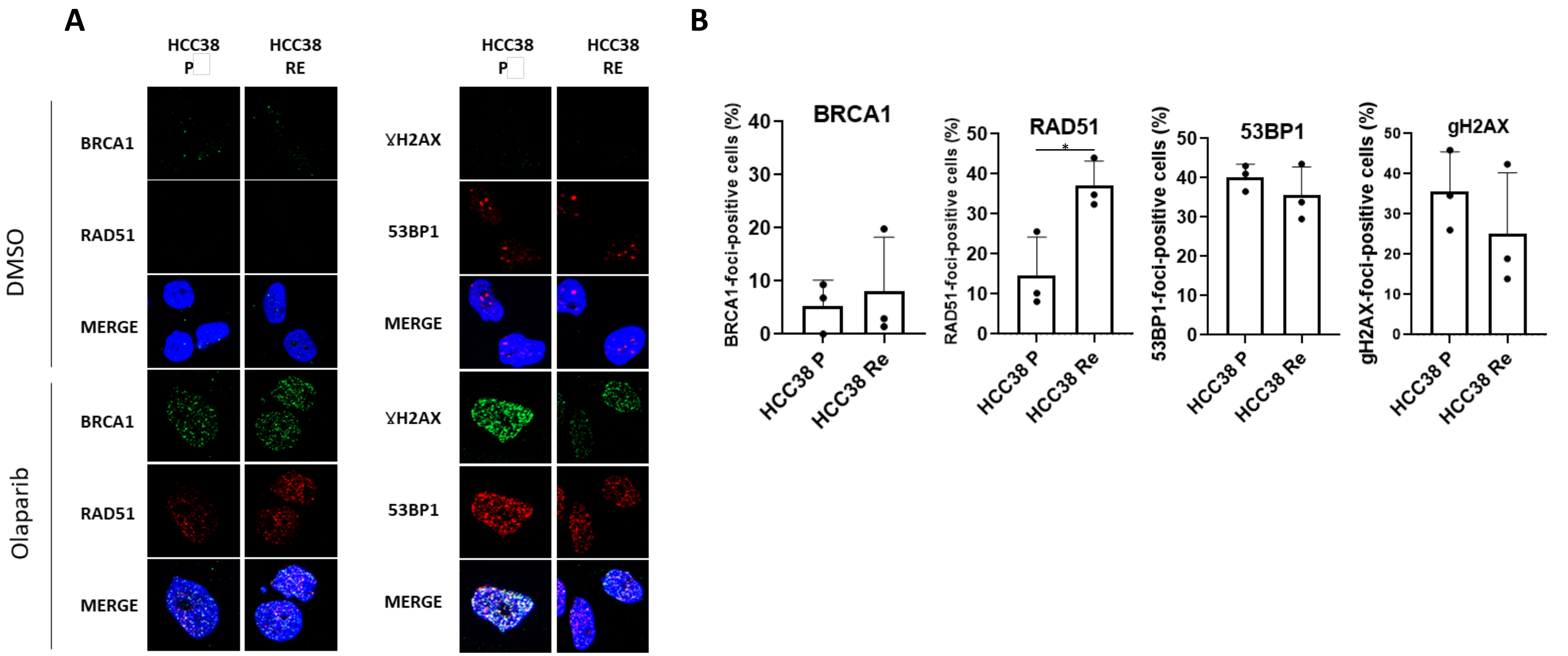

Supplementary Figure 7: A: **Olaparib IC50 levels (µM) of the different cell line models and derived Olaparib-resistant variants**. **A:** BRCA1, RAD51, gH2AX and 53BP1 nuclear foci in the *BRCA1* hemimethylated HCC38 parental cell line and olaparib resistant variant. **B:** BRCA1, RAD51, gH2AX and 53BP1 nuclear foci quantification.
