# Supplementary material for: BRCA1-methylated triple negative breast cancers previously exposed to neoadjuvant chemotherapy form RAD51 foci and respond poorly to olaparib
Source: Front Oncol. 2023 Mar 17;13:1125021. doi: 10.3389/fonc.2023.1125021 (PMC10064050; doi:10.3389/fonc.2023.1125021)
Supplement: Supplementary file 2 [file DataSheet_2.pdf]

Velazquez C, Ohran E. et al. Supplementary information

List of genes sequenced as part of the tNGS in TNBC PDX:

ABL1 ABL2 ACVR1B ADAMTS16 ADNP AFF2 AIP AJUBA AKAP9 AKT1 AKT2 AKT3
ALK ALKBH6 ANGPT2 ANK3 APC APOB AR ARHGAP35 ARID1A ARID1B ARID2
ARID5B ASXL1 ASXL2 ASXL3 ATM ATR ATRIP ATRNL1 ATRX AXIN1 AXIN2 AXL
BABAM1 BAP1 BARD1 BCLAF1 BCOR BLK BLM BMPR1A BRAF BRCA1 BRCA2 BRIP1
BUB1B C3ORF70 CACNA1S CAP2 CASP8 CBFB CBLB CCDC6 CCL2 CCND1 CCND2 CCND3
CCNE1 CCR4 CD22 CD248 CD33 CD38 CD3E CD3G CD4 CD40 CD52 CD70
CD74 CDC27 CDC73 CDH1 CDK12 CDK4 CDK6 CDKN1B CDKN1C CDKN2A CDKN2B
CDKN2C CEBPA CENPF CEP57 CHD4 CHEK1 CHEK2 CIC CMYA5 CNOT3 COL6A3 CRB1
CREBBP CSF1R CSMD3 CTCF CTGF CTLA4 CTNNA1 CTNNB1 CUL3 CUX1 CYLD DAXX
DDB2 DDR1 DDR2 DIAPH1 DICER1 DIS3L2 DLL4 DMD DNA2 DNER DNMT1
DNMT3A DNMT3B DSCAM DYNC1H1 ECT2L EGFL7 EGFR EIF1AX EIF2S2 ELF3 EME1 EME2
EP300 EPCAM EPHA2 EPHA3 EPHA5 EPHB2 ERBB2 ERBB2IP ERBB3 ERBB4 ERCC1 ERCC2
ERCC3 ERCC4 ERCC5 ESR1 EXO1 EXT1 EXT2 EZH2 EZR FAM123B FAM135B
FAM175A FANCA FANCB FANCC FANCD2 FANCE FANCF FANCG FANCI FANCL FANCM FAT1 FBXW7
FEN1 FES FGFR1 FGFR2 FGFR3 FGFR4 FGR FH FLCN FLT1 FLT3 FLT4
FNTA FOXA1 FOXP1 FOXQ1 FRK FUBP1 FYN FZD7 GALNT12 GATA1 GATA2 GATA3
GATA6 GEN1 GNAS GNPTAB GOT1 GPC3 GPNMB GPR149 GREM1 GRM1 H2AFX H3F3A
HCK HEATR5B HGF HNF1A HOXB13 HRAS HSPG2 HUS1 ICAM1 IDH1 IDH2 IGF1R
IGSF10 IL12A IL12B IL1B IL23A IL2RA IL2RB IL6R ING1 INO80 INPPL1 IPO7
IRF6 ITGA4 ITGAL ITGAV ITK JAK1 JAK2 JAK3 KDM5C KDM6A KDR KEAP1
KIT KLF4 KLHL8 KMT2A KMT2B KRAS L3MBTL3 L3MBTL4 LCK LCP1 LIG1 LIG3
LIG4 LRP1B LRRC1 LRRC7 LTK LYN MAD2L2 MADD MAP2 MAP2K1 MAP2K2
MAP2K4 MAP3K1 MAP3K10 MAP3K13 MAP3K4 MAP3K9 MAP4K3 MAPK1 MAX MBD1 MBTPS1 MDC1
MDM2 MDN1 MECOM MED12 MED23 MEN1 MET MGA MICALCL MINK1 MITF MLH1
MLH3 MLIP MLL3 MLLT4 MMS22L MORC4 MRE11A MS4A1 MSH2 MSH3 MSH6 MSLN
MST1R MTOR MTUS1 MUC1 MUS81 MUTYH MXRA5 MYB MYC MYCL1 MYCN MYH2
MYH9 NAV3 NBN NBPF1 NCAM1 NCOR1 NCOR2 NEB NF1 NF2 NFE2L2 NGF
NHEJ1 NKX2-1 NOTCH1 NOTCH2 NOTCH3 NOTCH4 NOV NR1I2 NRAS NSD1 NSMCE1
NSMCE2 NSMCE4A NTN4 NTRK1 NTRK2 NTRK3 OTUD7A PALB2 PARD3 PARP1 PARP2
PARP3 PARP4 PARP9 PBRM1 PCBP1 PCNA PDCD1 PDGFRA PDGFRB PHF6 PHOX2B PIF1
PIGF PIK3CA PIK3CB PIK3CD PIK3CG PIK3R1 PINK1 PLK1 PMS1 PMS2 POLA1 POLD1
POLD2 POLD3 POLD4 POLE POLQ PPM1D PPP2R1A PPP2R5C PRDM1 PREX2 PRF1
PRICKLE1 PRICKLE2 PRKAR1A PRKCB PRKCE PRKCG PRKCH PRKCQ PRKDC PRSS1 PSMB5
PSMC3IP PTCH1 PTCH2 PTEN PTK2 PTK7 PTPN11 PTPRD RAC1 RAD1 RAD17 RAD18
RAD50 RAD51 RAD51AP1 RAD51B RAD51C RAD51D RAD52 RAD54B RAD54L RAD9A RAD9B
RAF1 RASA1 RB1 RBBP8 RBM10 RECQL RECQL4 RECQL5 RET REV1 REV3L RFC1
RFC2 RFC3 RFC4 RFC5 RHBDF2 RHOA RIF1 RIT1 RNF43 RNF8 ROBO1 ROBO2
ROR1 ROR2 ROS1 RPA1 RPA2 RPA3 RPA4 RPL22 RPS6KC1 RSBN1L RTEL1 RUNX1
RUNX2 RUNX3 RXRA SACS SBDS SCRIB SDHA SDHAF2 SDHB SDHC SDHD SELP
SETD2 SF3B1 SGK1 SIRT1 SIRT4 SLAMF7 SLC1A3 SLC44A3 SLC4A5 SLIT2 SLX1A SLX4
SMAD2 SMAD4 SMARCA4 SMARCA5 SMARCAD1 SMARCAL1 SMARCB1
SMO SNX25 SOS1 SOX17 SOX9 SPEN SPOP SRC STAG2 STAT3 STAT5B STK11
STX2 SUFU TAF1 TAP1 TBC1D1 TBL1XR1 TBX3 TCF7L2 TCP11L2 TDP1 TEK TERT
TET1 TET2 TET3 TGFB2 TGFB3 TGFBR2 TMEM127 TNFRSF10A TNFRSF10B
TNFRSF11A TNFRSF12A TNFRSF8 TNFRSF9 TNFSF13B TP53 TP53BP1 TPX2 TRIM23
TRPC1 TSC1 TSC2 TTLL9 TXNDC8 U2AF1 UBE2N UIMC1 USH2A USP9X VANGL1
VANGL2 VEGFA VEGFB VHL WEE1 WRN WT1 XBP1 XPA XPC XRCC1 XRCC2
XRCC3 XRCC4 XRCC5 XRCC6 YES1 ZFHX3 ZFP36L1 ZHX2 ZMYND10 ZNF217 ZNF471 ZNF620
ZNF703 ZNF750 ZNRF3 ZRANB3

Gene	Forward	Reverse
BRCA1	TCCAAACCTGTGTCAAGCTG	CTTATTCCATTCTTTTCTCTCACACAG
BRCA1	GATTCTGCAAAAAAGGCTGCT	CAGATGCTGCTTCACCCTGA
BRCA2	TACAGTTGGCTGATGGTGGA	CCATAGCTGCCAGTTTCCAT
53BP1	TGGTTCCATCAGTCAGGTCA	CCTCAGCACCAAGGGAATGT
RAD51	CAGTGATGTCCTGGATAATGTAGC	TTACCACTGCTACACCAAACTCAT
RAD51C	GGTTCCAGACTGCTGAGGAA	TTATGAAGCCCTGGGTATGC
RAD51D	CAGGCTAAAACCCAGGATGA	CCACCACCTTCACAGTTCCT
PARG	AGGAGAGAGTCGCACTGGAA	CTGGAGAAAGGTGAGGTGGA
PALB2	CCCAGCATCAGATCATTGTG	ATGAAATGGAGCCGTGAAAG
RAD52	ACAGTGCCAGTACACAGCAGAAGA	GTGCCCAGCCATTGTAACCAAACA
ATM	CTGCAGAGAAACACGGAAAC	CCTGTGCACCATTCAAGAAC
ATR	CCAGGCATCCTCCTATTTTTC	TTTTCACCATGACGGTCTCC
PARP1	GAGTCGAGTACGCCAAGAGC	TCAGAGAACCCATCCACCTC
POLQ	CGTTCAGAATCAGGC	AAGTCCCCAGTTTGCCAATA
ABCB1	CCCATCATTGCAATAGCAGG	GTTCAAACTTCTGCTCCTGA
ABCC1	GGTCAGCCCAACTCTCTTGG	CACACACTAGGGCTACCAGC
ABCG2	GACTTATGTTCCACGGGCCT	TCTCTGTTTAATGCCACAGCA
GAPDH	TGCACCACCACCTGCTTAGC	GGCATGGACTGTGGTCATGAG

Supplementary Table 2: list of primers used in Q-RTPCR experiments. Sequences are presented 3' to 5'

	b1995	b3804	15b0018	15b1516	B3977	b4122	tm168	o10047	tm168
BRCA1	WT	WT	Me/Me	Me/UM	Me/Me	Me/Me	S1524fs	Del Ex 8-13	
BRCA2	WT	WT	WT	WT	WT	WT	WT	WT	
PALB2	WT	WT	WT	WT	WT	WT	WT	WT	
TP53	G329C	R280K	R213X	R273H	383delC	WT/LOH	R196X	L206fs	
RIF1	WT	WT	E279K	WT	WT	WT	WT	WT	
53BP1	WT	WT	WT	WT	WT	WT	WT	WT	
PTEN	285_293 del	WT	c1010_1026 del	WT	WT	WT	WT	WT	
KRAS	WT	G12C	Q61P	WT	WT	WT	WT	WT	
STK11	WT	G766K	WT	WT	WT	WT	WT	WT	

Supplementary Table 2 : Mutated and Hypermethylated DDR (DNA Damage Response) related genes in the TNBC PDX models tested in this study. Mutations at the *TP53* and *STK11* loci are indicated as amino acid changes. Those at the *BRCA1* and *PTEN* loci are indicated using the nucleotide position and associated modification, fs (frame shift) del (deletion). Wild type sequence is indicated as wt.

Target	Assay	Antibody Reference	Dilution Used	Supplier
BRCA1	IF	sc-6954	1/100	SCBT, Heidelberg, Germany
BRCA1	WB	9010	1/500	CST OZYME, Saint Cyr l'Ecole, France
BRCA2	WB	A303-434A	1/1000	CST OZYME, Saint Cyr l'Ecole, France
PARP1	WB	WH0000142M1	1/1000	Sigma Aldrich, Saint Quentin Fallavier, France
RAD51	IF	PC130	1/300	Merck Millipore Sigma Aldrich, Saint Quentin Fallavier, France
RAD51	WB	8875	1/1000	CST OZYME, Saint Cyr l'Ecole, France
gH2AX	IF	H2-3F4	1/4000	kind gift from Dr. Mustapha Oulad-Abdelghani, MAB-IGBMC Illkirch-Graffenstaden
53BP1	IF	NB100-304	1/500	Bio-techne LTD, Abington, UK
53BP1	WB	rabbit NB100 -304	1/2000	Bio-techne LTD, Abington, UK
Geminin	IF	52508	1/200	CST OZYME, Saint Cyr l'Ecole, France
Tubulin	WB	T9026	1/20000	Sigma Aldrich, Saint Quentin Fallavier, France
anti-mouse	IF	goat Alexa Fluor 488 ab150113	1/1000	Abcam, Cambridge, UK
anti-rabbit	IF	goat Alexa Fluor 555 ab150078	1/1000	Abcam, Cambridge, UK
anti-mouse	WB	HRP 70745	1/10000	CST OZYME, Saint Cyr l'Ecole, France
anti-rabbit	WB	HRP 7076	1/10000	CST OZYME, Saint Cyr l'Ecole, France

Supplementary Table 3 : list of the antibodies used in this study

Legends to the Supplementary Figures

Supplementary Figure 1: patient follow up data of the original tumors from which the PDX have been derived

Supplementary Figure 2: Methylation Specific (MS-PCR) analysis of the 4 PDX with BRCA1 promoter hypermethylation.

Supplementary Figure 3: Response to Olaparib and Carboplatin of the 8 PDX models. **A**; tumor growth curves in each experimental arm (vehicle, Olaparib, Carboplatin) were expressed as the mean of normalized tumor volume (TV). Carboplatin was administrated intraperitoneally at 50 mg/kg twice a week for 4 weeks. Olaparib was administrated by oral gavage at 100 mg/kg 5days/week for 5 weeks. **B**; PDX b3977, b4122 and 010047 were monitorited after end of treatment for tumor regrowth. Black circles and arrowhead indicate end of CBP and olaparib treatment respectively.

Supplementary Figure 4: copy number changes and chromosomal instability scores of the tested TNBC PDX models. **A**: copy number changes involving genes in the HR and NER pathways. Copy numbers determined using CGH-array Agilent arrays and expressed as Log2 of the fluorescence ratio of the Normal reference DNA and tumor DNA. Copy Numbers were infered using a diploid genome as reference.

Supplementary Figure 5: BRCA1, RAD51, γH2AX and 53BP1 nuclear foci in olaparib-treated PDX models. Data complementing results in Figure 3A

Supplementary Figure 6: **Quantification of Geminin positive cells in vehicle and Olaparib treated PDXs**. Frozen tissue sections from vehicle and olaparib treated PDX were sampled as described in Figure 3 and the fraction (%) of cells showing positive staining for Geminin a protein associated to DNA replication determined by immunofulorescence. Note that the fraction Geminin-positive cells remained stable or increased in most olaparib treated PDX.

Supplementary Figure 7: **BRCA1, RAD51, γH2AX and 53BP1 nuclear foci in Olaparib-treated cell line models**. **A**: Representative immunofluorescence images of indicated cell lines treated or not (control) with Olaparib at their respective IC50 concentrations for 24h. Cell line nomenclature is SUM159; *BRCA1*-WT (WT), BRCA1-KO (KO), BRCA1-KO-Ola-Resistant (Re), SUM149; parental (PT), Ola-Resistant (Re), UWB1.289; parental (PT), Ola-Resistant (Re)

Supplementary Figure 8: A: **Olaparib IC50 levels (µM) of the different cell line models and derived Olaparib-resistant variants**. **A:** BRCA1, RAD51, gH2AX and 53BP1 nuclear foci in the *BRCA1* hemimethylated HCC38 parental cell line and olaparib resistant variant. **B:** BRCA1, RAD51, gH2AX and 53BP1 nuclear foci quantification.
